# Identifying essential implementation strategies: a mixed methods process evaluation of a multi-strategy policy implementation intervention for schools

**DOI:** 10.1186/s12966-022-01281-5

**Published:** 2022-04-12

**Authors:** Cassandra Lane, Patti-Jean Naylor, Adam Shoesmith, Luke Wolfenden, Alix Hall, Rachel Sutherland, Nicole Nathan

**Affiliations:** 1 School of Medicine and Public Health, The University of Newcastle, 1 University Drive, Callaghan, NSW 2308 Australia; 2 Hunter New England Population Health, Hunter New England Area Health Service, Newcastle, NSW Australia; 3 Priority Research Centre for Health Behaviour, The University of Newcastle, Newcastle, NSW Australia; 4 Hunter Medical Research Institute, New Lambton Heights, NSW Australia; 5 School of Exercise Science, Physical and Health Education, University of Victoria, Victoria, BC Canada

**Keywords:** Physical activity, Policy, Implementation, Adaptations, Mixed methods, Scale-up, School, Children, Champions

## Abstract

**Background:**

Physically Active Children in Education (PACE) is composed of eight implementation strategies that improves schools’ implementation of a government physical activity policy. A greater understanding of each discrete implementation strategy could inform improvements to PACE for delivery at-scale. This study aimed to: (A) measure the dose delivered, fidelity, adoption and acceptability of each strategy using quantitative data; (B) identify implementation barriers and facilitators using qualitative data; and (C) explore the importance of each strategy by integrating both data sets (mixed methods).

**Methods:**

This study used data from a cluster randomised noninferiority trial comparing PACE with an adapted version (Adapted PACE) that was delivered with reduced in-person external support to reduce costs and increase scalability. Data were collected from both trials arms for between-group comparison. Descriptive statistics were produced using surveys of principals, in-school champions and teachers; and project records maintained by PACE project officers (objective A). Thematic analysis was performed using in-school champion and project officer interviews (objective B). Both data sets were integrated via a triangulation protocol and findings synthesized in the form of meta-inferences (objective C).

**Results:**

Eleven in-school champions and six project officers completed interviews; 33 principals, 51 in-school champions and 260 teachers completed surveys. Regardless of group allocation, implementation indicators were high for at least one component of each strategy: dose delivered =100%, fidelity ≥95%, adoption ≥83%, acceptability ≥50%; and several implementation barriers and facilitators were identified within three broad categories: external policy landscape, inner organizational structure/context of schools, and intervention characteristics and processes. All strategies were considered important as use varied by school, however support from a school executive and in-school champions’ interest were suggested as especially important for optimal implementation.

**Conclusion:**

This study highlights the importance of both executive support and in-school champions for successful implementation of school physical activity policies. In particular, identifying and supporting an in-school champion to have high power and high interest is recommended for future implementation strategies. This may reduce the need for intensive external support, thus improving intervention scalability.

**Supplementary Information:**

The online version contains supplementary material available at 10.1186/s12966-022-01281-5.

## Background

Due to the complexity of many public health interventions, it is notoriously difficult to uncover underlying mechanisms surrounding an intervention’s effect or lack thereof. Process evaluations address this ‘black box’ of effectiveness [1] by providing important information to help explain how and why outcomes occurred [1–3]. As such, conducting process evaluations alongside intervention effectiveness trials is recommended [1, 3, 4]. The findings may reduce research waste; save valuable time and resources of health service providers; and provide decision makers with direction, particularly for intervention scale-up and dissemination in other contexts [3, 5].

Implementation interventions aiming to enhance the uptake of evidence-based policies or practices are often quite complex [6]. Although some interventions use only one or two strategies to assist implementation, many consist of multiple strategies that target numerous implementation determinants [7]. To date, there are over 70 discrete implementation strategies documented [8], each of which is multifaceted in nature [6]. For example, the single strategy ‘conduct educational meetings’ may involve more than one stakeholder group, mode of delivery, setting and/or occurrence. These complexities pose a challenge determining which strategy, or combination of strategies, most likely led to the outcomes of implementation intervention studies (and how). Given that implementation science is a relatively new area of science, little is known about the most efficient and effective approaches to achieve implementation of evidence-based policies or practices in various contexts [7]. Robust process evaluations of packaged implementation strategies hold considerable potential to contribute to this scant evidence base.

This paper describes the mixed methods process evaluation undertaken as part of a research initiative optimising a package of implementation strategies to improve schools’ implementation of a government physical activity policy. In response to poor physical activity policy implementation by schools worldwide [9–19], and limited research about strategies to support schools to address this, Nathan et al. developed the *‘Physically Active Children in Education (PACE)’* intervention [20]. PACE consisted of eight implementation strategies, each chosen using the Theoretical Domain Framework (TDF) and the Behaviour Change Wheel (BCW) to overcome identified barriers [21], to support school’s compliance with an Australian state-level physical activity policy [22]. In a series of randomised and controlled trials (RCTs), PACE consistently improved policy implementation by schools [17, 23] alongside high reports of fidelity and perceived satisfaction by stakeholders [17]. Furthermore, its meaningful impact was maintained at a lower cost to the health service provider when several implementation strategies were adapted to reduce the in-person contact time used to support schools (i.e., Adapted PACE) [24]. Although effective, little was known about which PACE implementation strategies, in either their original or adapted format, were *“most commonly needed, feasible to deploy, and effective across implementation efforts*” [25]. A greater understanding of each implementation strategy could inform further optimisation processes [26, 27] to improve PACE for delivery to the remaining 400+ schools in the health service region [28] and for broader scale-up. Additionally, this information could contribute to a scant evidence base regarding discrete implementation strategies employed both in schools and other settings.

We conducted a mixed methods process evaluation alongside a cluster randomised implementation trial to explore the implementation of the eight discrete PACE implementation strategies from the perspective of school stakeholders and PACE delivery personnel. This included a comparison of PACE and Adapted PACE, to highlight any qualitative differences when delivered with reduced in-person support. The specific objectives were:

A. To quantitatively measure each strategy in regards to key implementation indicators including dose delivered, fidelity, adoption and acceptability (implementation outcomes [29]);
B. To qualitatively explore factors that influenced program implementation (barriers and facilitators); and
C. To assess the importance of each strategy using outcomes from both the quantitative and qualitative data sets.

## Methods

### Research design

This process evaluation accompanies the Adapted PACE cluster randomised noninferiority trial [24] undertaken from October 2018 – December 2019 (Australian New Zealand Clinical Trials Registry: ACTRN12619001229167). We used a convergent mixed methods triangulation research design with quantitative and qualitative data from participants in both active trial arms collected simultaneously and weighted equally (QUAN+QUAL) [30, 31]. The Medical Research Council (MRC) guidance on the process evaluations of complex interventions [3], and the evaluation roadmap for physical activity interventions developed by McKay et al. [29], informed study design, planning and execution.

### The Adapted PACE noninferiority trial

Full details of the Adapted PACE cluster randomised noninferiority trial is published elsewhere [24]. Briefly, the trial was conducted in 48 primary schools in NSW, Australia. Following consent and completion of baseline data collection, an independent statistician used a computerised random number function to block randomise schools in a 1:1 ratio to receive either PACE or Adapted PACE. Table 1 includes an overview of the eight implementation strategies (listed numerically) and their components (alphabetised), and adaptations made for Adapted PACE. In short, strategy 1 (ongoing support for the in-school champion [ISC]) and 2a (principal meeting) were delivered via email/telephone rather than in-person, and strategy 5 (educational outreach visit) was delivered by an ISC rather than an external project officer.

**Table 1:** Description of PACE implementation strategies and the joint display of quantitative implementation indicators (adoption and acceptability), usefulness and relevant qualitative themes for each

Strategy	Description	Adoption	Acceptability	Usefulness/importance for program implementation
1. Centralise technical assistance and provide ongoing consultation with one or more experts in the strategies used to support implementing the innovation (External ongoing support)	Project officers (a PE teacher and health promotion practitioner) employed by the health service provided technical assistance to ISC throughout the study period (12 months) in-person and/or email/telephone. Their role was to provide expertise, advice and resources to ISC to help them problem solve barriers to policy implementation. Adapted PACE: via distance correspondence only (email/telephone)	ISC (the primary source of contact) from each school engaged in communication with, and support offered by, project officers *(project records)*: 47/48 (98%) - PACE: 23/24 (96%) - Adapted PACE: 24/24 (100%)	The support I received from the project officer to implement PACE at my school was adequate *(ISC)*: 40/46 (87%) - PACE: 16/18 (89%) - Adapted PACE: 24/28 (86%) *23/28 (82%) indicated that support received from the project officer to deliver the teacher education session was adequate.	This was not ranked within the top 3 for ‘most important strategy’ by any project officers. *Qualitative themes:* (i) An engaging innovation via: Project officer characteristics [facilitator]; (ii) Limited time/competing demands of staff [barrier].
2. Mandate change	2a. Project officers had one × 1 h meeting, in-person and/or by email/telephone, with school principals and school executives to communicate the importance and benefits of the policy and gain their commitment for policy implementation over the school year. Adapted PACE: via distance correspondence only (email/telephone)	Schools with principals that participated in the meeting and provided verbal commitment *(project records)*: 48/48 (100%) - PACE: 24/24 (100%) - Adapted PACE: 24/24 (100%)	N/A	This was considered the most important strategy (ranked first) by 5/6 project officers. *Qualitative themes:* (i) Supportive executive [facilitator]; (ii) School physical activity culture [facilitator]; and (iii) ISC power**-**interest - Player [facilitator], Context Setter [barrier], Subject or Crowd Member [barrier]. Having principal/executive support to schedule physical activity was useful/extremely useful: *(ISC)*: 37/44 (84%) - PACE: 16/XX (94%) Adapted PACE: ISC: 21/X (79%)
	2b. School executives were asked to demonstrate their commitment to implementing the NSW policy through the development of a school-level policy (‘Sport and Physical Activity Procedures document’) as required by the state-level policy.	Principals that indicated their school had a physical activity policy *(principals)*: 13/33 (39%) - PACE: 5/15 (33%) - Adapted PACE: 8/18 (44%) Principals that indicated their school had a physical activity policy – existing or in development *(principals)*: 26/33 (79%) - PACE: 12/15 (80%) - Adapted PACE: 14/18 (78%)	N/A	
	2c. Principals and school executives were asked to demonstrate their support for, and the importance of, the policy by communicating to the broader school community (e.g., via newsletters, assemblies and staff meetings) that the implementation of the policy was a school priority and expected of all staff.	Principals that communicated their support (full or partial) for the implementation of the policy to teachers *(principals)*: 27/32 (84%) - PACE: 13/14 (98%) - Adapted PACE: 14/18 (78%) Principals that indicated PACE was a school priority *(principals)*: 23/32 (72%) - PACE: 10/14 (71%) - Adapted PACE: 13/18 (72%) Teachers that agreed/strongly agreed they had support from their school executive to implement PACE *(teachers)*: 161/229 (70%) - PACE: 67/94 (71%) - Adapted PACE: 94/135 (70%)	N/A	
3. Identify and prepare champions	3a. Each school nominated one to two ISC (existing teacher(s) at the school) who, with the support of the project officer, were responsible for leading the development and implementation of the policy in the school over the next 12 months.	Teachers that agreed/strongly agreed they had support from their ISC to implement PACE *(teachers)*: 137/182 (75%) - PACE: 57/76 (75%) - Adapted PACE: 80/106 (75%)	The assistance I received from my ISC was acceptable *(teachers)*: 126/173 (73%) - PACE: 48/69 (70%) - Adapted PACE: 78/104 (75%)	This was considered an important strategy (top three) by 100% of project officers; one of which ranked it first most important. *Qualitative themes:* (i) ISC power-interest – Player [facilitator], Context Setter, Subject or Crowd Member [barrier]; (ii) An engaging innovation via: experiential learning [facilitator] and project officer characteristics [facilitator]; and (iii) Adaptability/flexibility of PACE: a choice-based model [facilitator]. Having an ISC located within the school was useful/extremely useful: *(ISC)*: 35/45 (78%) PACE: 13/17 (76%) Adapted PACE: 22/28 (79%)
	3b. ISC attended 1 × full day (5 h) training workshop with ≤20 ISC from other schools. These workshops covered: education about the policy, instruction and demonstration of energisers, active lessons and PE lessons, time to begin action planning, identification of barriers/ facilitators to implementation and possible solutions to overcome these. The training was accredited by the state educational authority and provided teachers continuing professional development hours.	ISC that actively participated in the workshop education and activities *(project records)*: 80/80 (100%) - PACE: 38/38 (100%) - Adapted PACE: 42/42 (100%)		
4. Develop a formal implementation blueprint	ISC were supported to develop a plan for policy implementation in their school. The plan identified what the school was aiming to achieve, the strategies to do so and by when, and the resources available or required. The plan was segmented into school terms to break up the more complex policy requirements into achievable tasks.	ISC were provided with time to complete this strategy during strategy 3b, the ISC workshop *(project records)*: 100%	The physical activity plan developed by the ISC was acceptable in assisting me to schedule physical activity in my class *(teachers)*: 114/172 (66%) - PACE: 41/69 (59%) - Adapted PACE: 73/103 (71%)	This was not ranked within the top 3 for ‘most important strategy’ by any project officers; however it was noted as occurring as part of the ISC workshop (strategy 3b). *Qualitative themes:* (i) An engaging innovation via quality resources [facilitator]; and (ii) Staff turnover [barrier]. The creation of the school physical activity policy, scope and sequence document and whole school physical activity timetable was useful/extremely useful: *(ISC):* 34/44 (77%) - PACE: 14/17 (82%) - Adapted PACE: 20/27 (74%)
5. Conduct educational outreach visits	School staff attended 1 × 1–2 h information and training session delivered by an external representative (led in-person by a project officer) during a school staff meeting. Teachers were provided with an overview of the policy, the importance of and requirements for its implementation. The plan for their schools’ implementation of the policy was presented including the timeline of when key milestones were expected. They participated in practical demonstrations of suggested physical activities (e.g., energisers and active lessons) which they could incorporate into their normal classroom routines. Adapted PACE: peer-delivered by ISC	Schools in which staff participated in the staff information and training session *(project records):* 40/48 (83%) - PACE: 23/24 (96%) Adapted PACE: 17/24 (71%) *3 unknown	Overall: The whole school staff meeting was acceptable in assisting me to schedule physical activity in my class *(teachers)*: 126/176 (72%) - PACE: 59/72 (82%) - Adapted PACE: 67/104 (64%) Content: The information provided at the whole school meeting was acceptable in assisting me to schedule physical activity in my class *(teachers)*: 125/176 (71%) - PACE: 58/72 (81%) - Adapted PACE: 67/104 (64%) (As above) Adapted PACE schools only: The support I received from the project officer to deliver the teacher education session was adequate (*ISC*): 23/28 (82%)	This was considered an important strategy (top three) by 4/6 of project officers. *Qualitative themes:* (i) Teachers’ attitudes, beliefs and level of support [implementation facilitator]; (ii) Adaptability/flexibility of PACE: a choice-based model [facilitator]; (iii) An engaging innovation via experiential learning [facilitator]; (iv) Limited time/competing demands of staff [barrier]; (v) Staff turnover [barrier]; (vi) Adapted PACE only - ISC power-interest – Player [facilitator], Context Setter, Subject, or Crowd Member [barrier]; and (vii) PACE only - An engaging innovation via: project officer characteristics [facilitator]. PACE schools only: Having the project officer conduct the one-hour educational outreach visit was useful/extremely useful *(ISC survey):* 15/17 (88%)
6. Develop and distribute educational materials	6a. ISC received an “intervention manual” inclusive of policy templates as well as examples of a physical activity timetable and PE curriculum.	ISC were provided with these materials during strategy 3b, the workshop *(project records)*: 100%	The information on the online portal was acceptable *(teachers)*: 74/173 (43%) - PACE: 33/70 (47%) - Adapted PACE: 41/103 (39%)	Resources were considered an important strategy (top three) by 2/6 of project officers. *Qualitative theme:* (i) An engaging innovation via quality resources [facilitator]. Responses of ‘yes’ - the resources available through the online portal were useful in helping to schedule physical activity *(teachers)*: 97/150 (65%) PACE: 45/62 (73%) Adapted PACE: 52/88 (59%)
	6b. ISC and teachers received educational materials during their respective training sessions. These resources included ideas and strategies for practical games that increase physical activity during class time, example timetables etc.	PACE resources: Teachers’ that used PACE resources (e.g., online portal, booklet and equipment pack) to help schedule physical activity *(teachers)*: 118/202 (58%) - PACE: 56/86 (65%) - Adapted PACE: 62/116 (53%) Online portal (schools): Schools with at least one teacher that accessed the online portal *(project records: web analytics)*: 43/48 (90%) - PACE: 22/24 (92%) - Adapted PACE: 21/24 (88%) Online portal (teachers): Teachers that accessed the resources on the online portal *(teachers)*: 98/228 (43%) - PACE: 42/95 (44%) - Adapted PACE: 56/133 (42%)		
	6c. ISC and teachers were provided access to professional learning videos which reinforced information received during their respective training sessions. ISC were asked to view the videos and to organise a time for their colleagues to watch them during a staff meeting.	Schools in which at least one professional online learning video was viewed by teachers and ISC *(project records: web analytics)*: Teachers: 24/48 (50%) - PACE: 11/24 (46%) - Adapted PACE: 13/24 (54%) ISC: 35/48 (80%) - PACE: 12/24 (50%) - Adapted PACE: 23/24 (82%)	(As above) The information on the online portal was acceptable *(teachers)*: 74/173 (43%) - PACE: 33/70 (47%) - Adapted PACE: 41/103 (39%)	
7. Capture and share local knowledge (success stories)	ISC and teachers were provided access to case studies from other schools. Case studies described “success stories” of how ISC and teachers had overcome frequently reported barriers to implement the policy in their school.	Schools in which at least one teacher accessed the online portal *(project records: web analytics)*: 43/48 (90%) - PACE: 22/24 (92%) - Adapted PACE: 21/24 (88%)	(As above) The information on the online portal was acceptable *(teachers)*: 74/173 (43%) - PACE: 33/70 (47%) - Adapted PACE: 41/103 (39%)	This was not ranked within the top 3 for ‘most important strategy’ by any project officers.
8. Change physical structure and equipment (equipment pack)	8a. Each school was provided with a physical activity pack consisting of items to engage in suggested activities (i.e., activities advertised in educational materials and/or exemplified during the ISC workshop and teacher training) such as bean bags, balls, hoops, etc.	ISC were provided an equipment pack during strategy 3b, the workshop *(project records)*: 100% (As above) Teachers’ that used PACE resources (e.g., online portal, booklet and equipment pack) to help schedule physical activity *(teachers)*: 118/202 (58%) - PACE: 56/86 (65%) - Adapted PACE: 62/116 (53%)	The equipment pack was acceptable in assisting me to schedule physical activity in my class *(teachers)*: Total: 123/217 (57%) - PACE: 57/88 (65%) - Adapted PACE: 66/129 (51%)	Providing schools with an equipment pack was considered an important strategy (top three) by 2/6 of project officers. *Qualitative theme:* (i) An engaging innovation via quality resources [facilitator]
	8b. ISC were encouraged to develop classroom physical activity packs for all teachers using existing school sport equipment. These packs were to be kept in each classroom, enabling teachers to implement suggested activities more easily.	ISC that purchased equipment packs (ful or partial) for all classrooms *(ISC)*: 34/44 (77%) - PACE: 13/16 (81%) - Adapted PACE: 9/28 (32%)		

*Abbreviations*: *ISC* In-school champion

*Note*: Quantitative data retrieved from project records (project records), ISC surveys (ISC), principal surveys (principals), and teacher surveys (teachers)

Consistent with the previous PACE trials [17, 23], the primary outcome measure used to assess program impact was teachers’ mean minutes of total physical activity (physical education [PE], sport, energisers or active lessons) implemented across the school week. Both groups saw improvements in the minutes of physical activity teachers implemented per week at follow-up, and there was a high probability of no meaningful difference between the effect of original PACE and Adapted PACE. A cost minimisation analysis conducted from the health service provider perspective showed an estimated reduction (in AUD) of $373 (Uncertainty Interval = $178, $573) per school to deliver Adapted PACE compared to PACE. On the basis of these findings, we concluded that Adapted PACE is a cost-efficient alternative that is “as good as” [32] PACE in assisting schools’ implementation of a physical activity policy.

### Data collection

**Fig. 1:**
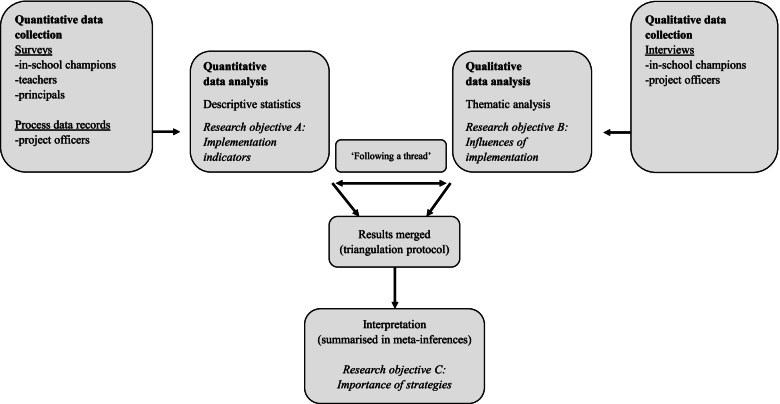
An overview of the convergent mixed method analytic procedure used for this study, including method of data collection and analysis to address each research objective. Quantitative data collection measures and method of analysis for research objective A is displayed on the left. Qualitative data collection measures and method of analysis for research objective B is displayed on the right. Both sides converge for the mixed methods procedure and interpretation for research objective C

To ensure a full range of perspectives, we sought to obtain data from all parties involved in the delivery and implementation of strategies: PACE project officers (a PE teacher and health promotion practitioner; schools’ primary source of contact) and school stakeholders targeted by strategies (principals, ISC and teachers). Quantitative data were collected from project records maintained by project officers throughout the study period and surveys of school stakeholders conducted immediately following intervention delivery at 12-month follow-up (Oct-Dec 2019). Qualitative data were collected via semi-structured interviews of project officers and a subsample of ISC also at 12-month follow-up. Figure 1 provides an overview of data collection methods and corresponding analytic procedures used to address each research objective.

#### Project records

Project officers documented the implementation of strategies using an Excel spreadsheet modified from previous trials of PACE [17, 20]. This included the expended time and mode of delivery (in-person, email or telephone) they employed to deliver strategies, as well as the school-level response such as staff engagement and attendance at training. These records enabled assessment of implementation outcomes (research objective A) as defined by McKay et al. [29], including dose delivered (*intended units delivered*), fidelity (*the extent to which strategies were implemented as prescribed*) and adoption (*proportion and representativeness of school stakeholders that utilised strategies)* (Additional file 1). School characteristics were also recorded to provide contextual information.

#### School stakeholder surveys

All consenting school stakeholders completed a paper survey modified from previous trials [17, 20]. In this study, these surveys were used to obtain respondent characteristics, inform implementation indicators [29] (research objective A) and assess usefulness of strategies (research objective C). Additional file 1 provides an overview of the measures used to assess the different indicators for each strategy. Briefly, principal surveys included measures of adoption for schools’ mandated change (e.g., if their school had a physical activity policy). Teacher surveys included measures of acceptability (e.g., the extent to which respondents agreed that the staff training was acceptable in assisting them to schedule physical activity); adoption (e.g., whether they had used PACE resources); and usefulness (e.g., whether the resources provided on the online portal were useful). ISC surveys were an expansion of the teacher survey, and included measures of usefulness for select strategies, inquiring the extent to which they were perceived as ‘useful’ for program implementation.

#### Semi-structured interviews of project officers

All project officers involved in the delivery of PACE (*N* = 6) were interviewed using a semi-structured interview guide developed following guidelines [31]. Open-ended questions explored project officer’s perspectives of program implementation by schools, including any barriers and facilitators to doing so (research objective B). Project officers were also asked to rank the top three strategies that they perceived as essential for successful program implementation and provide a rationale for these choices (research objective C). The facilitator used prompts to explore differences between PACE and Adapted PACE.

#### Semi-structured interviews of ISC

We purposively sampled ISC using a maximum variation approach [33] with a minimum target sample size of eight ISC: four from PACE schools and four from Adapted PACE schools with at least one in each group representing low and high levels of program engagement. Following recommendations for qualitative sampling [33, 34], project officers carried out recruitment by email invitation until we reached data saturation (i.e., sampling to the point of redundancy) to ensure sufficient depth of data for a robust analysis. Interviews offered in-person or by telephone were scheduled in advance with consenting ISC. They were conducted using a semi-structured interview guide developed following guidelines [31]. Open-ended questions explored participant’s experiences with PACE and factors that influenced implementation by schools (research objective B). Each participant received a $30 grocery gift card as a token of appreciation for their time.

### Data analysis

Quantitative and qualitative data were analysed separately and then merged for interpretation as per Creswell et al. [30] (Fig. 1).

#### Quantitative data analysis

An independent statistician used project records and survey data to produce descriptive statistics (mean, SD and proportions) to address each research objective.

#### Qualitative data analysis

Interviews were audio-recorded and transcribed verbatim. Interview facilitators checked transcripts for accuracy, corrected as necessary and anonymised any identifiable comments. The final copies were entered into QSR NVivo [35] for inductive thematic analysis using the standard approach reported by Braun and Clarke [36]. First, two experienced members of the research team (i) independently coded a subset of transcripts, (ii) checked for agreeance and resolved discrepancies with a third researcher, and (iii) developed a combined code scheme. One of the researchers then applied the code scheme to the remaining transcripts, discussing new codes with the other researcher and updating where necessary. The analysis pair then looked for patterns within the final codes and generated a list of themes that emerged in relation to research objective B. The proposed list of themes was reviewed, modified where required and finalised with names and definitions for each. This process was guided by a consensual qualitative research process [37] inclusive of ongoing discourse and confirmation with other members of the PACE evaluation and delivery team. In addition, one member of the research team searched project officer transcripts and recorded for each, the top three strategies reported as essential for successful program implementation (research objective C).

#### Mixed methods analysis

Quantitative and qualitative data were weighted equally due to their shared contribution in addressing evaluation objectives. Each data set were integrated [30, 38] using common methods for data integration. In the first method, known as ‘following a thread’ [38], we used preliminary findings from each data set to form hypothesis warranting further exploration, identify key themes, and/or interpret the other data set. In the second method of data integration, we compared and integrated the findings from each data set according to ‘triangulation protocol’ [38]. Specifically, we developed a side-by-side joint display table [39] with quantitative and qualitative findings juxtaposed in relation to each implementation strategy, facilitating cross-data comparison and convergence including areas of agreement, dissonance, or silence (when a theme from one data set is not found in another) [38]. We reported an integrative review of results derived from this process in the form of theoretical statements referred to as meta-inferences [40].

## Results

**Fig. 2:**
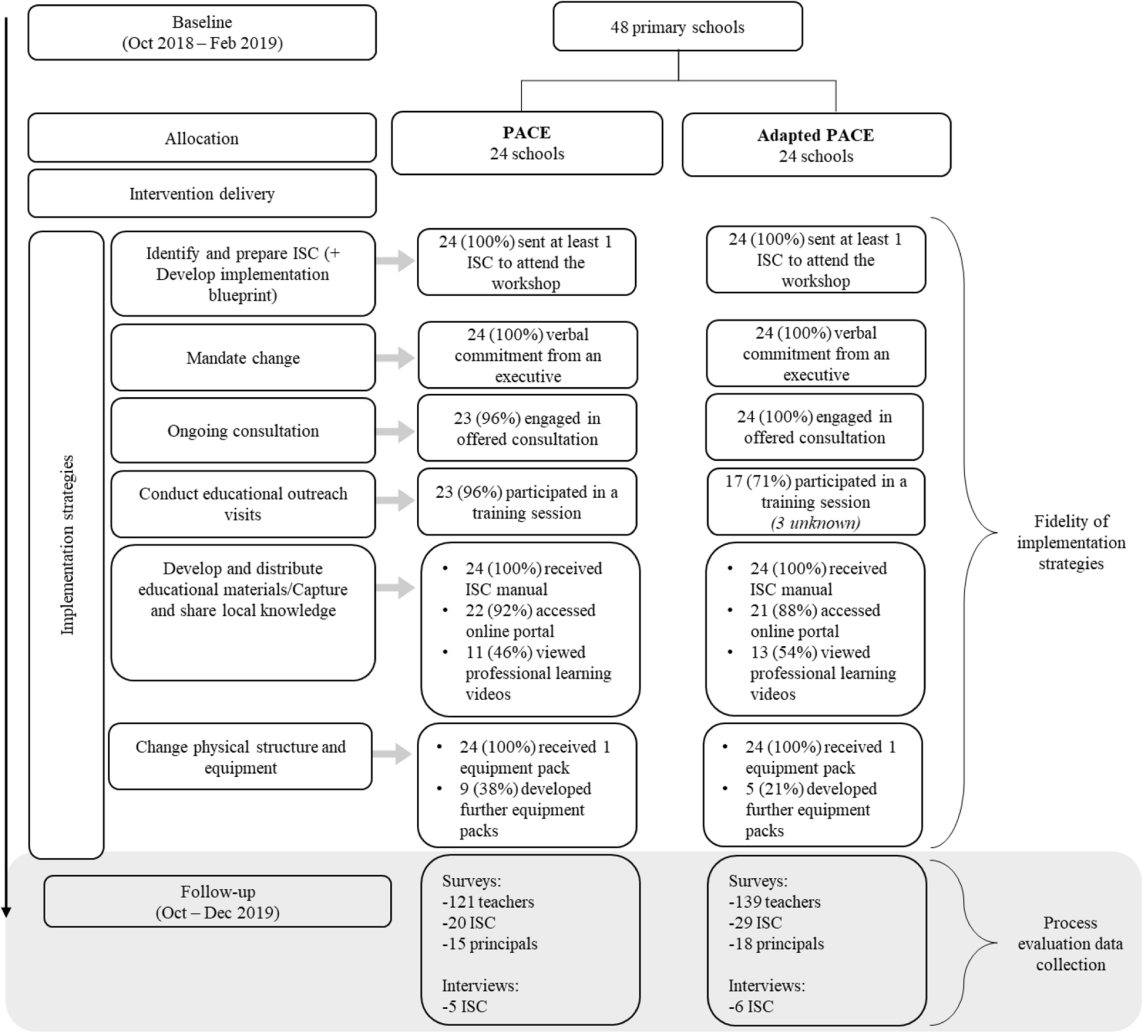
An overview and timeline of the PACE noninferiority trial [24] process evaluation. This shows when the process evaluation data was collected (at follow-up) in relation to the entire research trial timeline. It also shows schools’ fidelity of each implementation strategy – listed in order of delivery – for each of PACE and Adapted PACE schools

At 12-month follow-up, a total of 33 principals (33/48; response rate = 69%), 51 ISC (51/57 from 41 schools [some schools had > 1 ISC]; response rate = 89%) and 260 teachers (across all 48 schools) completed surveys; and 11 ISC took part in an interview (Fig. 2). Table 2 provides an overview of the characteristics of schools, principals and teachers, including a breakdown between groups.

**Table 2:** Overview of school, principal and teacher characteristics at 12-month follow-up

**School characteristics**	**PACE** **(*N*** **= 24)**	**Adapted PACE** **(*N*** **= 24)**	**Total** **(*N*** **= 48)**
School type			
• Catholic	2 (8%)	3 (13%)	5 (10%)
• Government	21 (88%)	20 (83%)	41 (85%)
• Independent	1 (4%)	1 (4%)	2 (4%)
Number of students (size)			
• Mean (SD)	205.9 (199.85)	242 (252.27)	224 (225.9)
• Median (range)	141 (7–819)	117 (11–768)	137 (7–819)
SEIFA^a^			
• Most disadvantaged	17 (71%)	16 (67%)	33 (69%)
• Least disadvantaged	7 (29%)	8 (33%)	15 (31%)
Geolocation			
• Major city	11 (46%)	11 (46%)	22 (46%)
• Inner/outer regional or remote	13 (54%)	13 (54%)	26 (54%)
**Principal characteristics**	**PACE** **(*N*** **= 15)**	**Adapted PACE (*N*** **= 18)**	**Total** **(*N*** **= 33)**
School type teaching at			
• Catholic	1 (7%)	1 (6%)	2 (6%)
• Government	14 (93%)	15 (83%)	29 (88%)
• Independent	0	2 (11%)	2 (6%)
Age	*N = 16*	*N = 18*	*N = 31*
• Mean (SD)	45.33 (10.32)	48.75 (9.17)	47.10 (9.73)
Sex			
• Female	9 (60%)	14 (78%)	23 (70%)
Years in role	*N = 14*	*N = 18*	*N = 32*
• Mean (SD)	3.08 (4.27)	3.83 (3.28)	3.5 (3.7)
Teaching principal	*N = 10*	*N = 10*	*N = 20*
• yes	4 (40%)	4 (40%)	58 (40%)
**Teacher characteristics**	**PACE** **(*N*** **= 121)**	**Adapted PACE (*N*** **= 139)**	**Total** **(*N*** **= 260)**
School type teaching at			
• Catholic	19 (7%)	6 (4%)	15 (6%)
• Government	94 (78%)	112 (81%)	206 (79%)
• Independent	18 (15%)	21 (15%)	39 (15%)
Age	*N = 91*	*N = 121*	*N = 212*
• Mean (SD)	39.19 (11.74)	40.66 (10.85)	40.03 (11.24)
Sex	*N = 102*	*N = 137*	*N = 239*
• Female	88 (86%)	110 (80%)	198 (83%)
Employment status	*N = 98*	*N = 137*	*N = 235*
• Full-time	87 (89%)	118 (86%)	205 (87%)
• Part-time/casual	11 (11%)	19 (14%)	30 (13%)
Years teaching experience	*N = 98*	*N = 134*	*N = 232*
• Mean (SD)	12.09 (9.68)	15.72 (11.49)	14.19 (10.98)
Specialist PE teacher	*N = 98*	*N = 135*	*N = 233*
• yes	4 (4%)	2 (1%)	6 (3%)
In-school champion			
• yes	21 (17%)	30 (22%)	51 (20%)

^a^Socio-Economic Indexes for Areas (relative socio-economic advantage and disadvantage)

### Research objective A: implementation indicators

#### Dose delivered and fidelity

Project officers delivered each strategy to all schools (dose delivered = 100%). Fig. 2 details fidelity of each implementation strategy. There was at least 95% fidelity for at least one component of each strategy at all schools with the exception of 71% fidelity of the educational outreach visit at Adapted PACE schools.

#### Adoption and acceptability

Table 1 includes a comprehensive description of the adoption and acceptability of strategies by targeted stakeholders. Overall the PACE implementation strategies were highly adopted, with no less than 83% adoption by schools and/or stakeholders to at least one component of each strategy. Some strategy components had low adoption (≤50%), in particular strategy 2b (school executives develop a school physical activity policy) with 39% of principals indicating an existing physical activity policy at their school. Other components with low adoption were those related to the online portal. For example, 50% of schools had at least one teacher view the professional learning videos available on the online portal and 43% of surveyed teachers reported accessing the online portal resources. There was little variation between PACE and Adapted PACE in regards to strategy adoption. Adapted PACE had 25% lower adoption to strategy 5 (staff training session) although three schools were ‘unknown’, and 49% lower adoption to strategy 8b (ISC develop further equipment packs).

The implementation strategies were considered highly acceptable by stakeholders. More than 50% of teachers agreed or strongly agreed that all but one strategy were acceptable in assisting them to schedule physical activity. The information on the online portal was considered acceptable by 43% of surveyed teachers. There was little variation between PACE and Adapted PACE schools in regards to strategy acceptability, with the exception of strategy 5 (staff training session), with nearly 20% fewer teachers at Adapted PACE schools indicating acceptability for both the overall session and content.

### Research objective B: influences of implementation

**Fig. 3:**
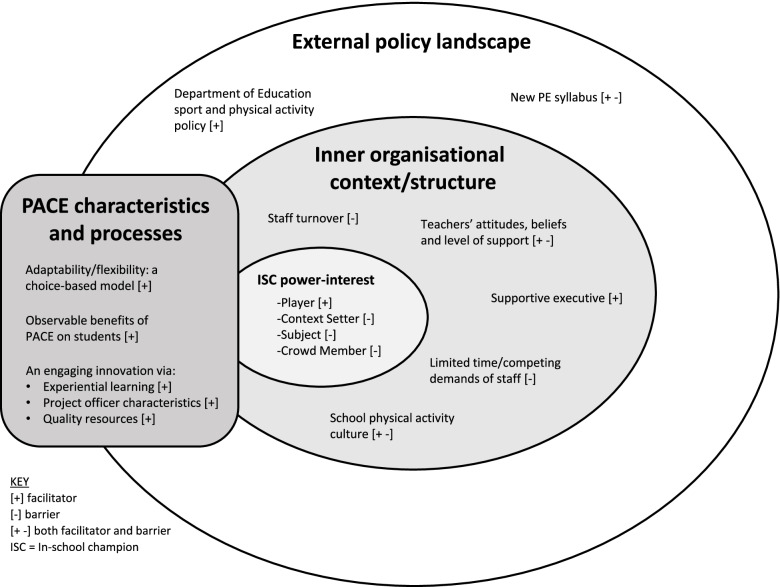
An overview of the influential factors of implementation that emerged through thematic analysis, falling within three broad categories: external policy landscape, inner organisational context/structure of schools, and PACE characteristics and processes

Thematic analysis revealed several influential factors of implementation (facilitators and barriers). Each theme fell within one of three identified categories: external policy landscape, inner organisational context/structure (schools) or PACE characteristics and processes. This fits within three of the five broad domains of the Consolidated Framework for Implementation Research (CFIR): outer setting, inner setting, and intervention characteristics [41]. Figure 3 provides an overview of themes and subthemes that emerged within each category, and Table S3 describes each and includes sample quotes.

#### External policy landscape

The external policy landscape consisted of the NSW Department of Education Sport and Physical Activity Policy [22], and the Personal Development, Health and Physical Education (PDHPE) syllabus released by NSW Education Standards Authority (NESA) in 2018 [42]. For the most part, these government mandates appeared to facilitate program implementation by necessitating that schools provided physical activity. The new syllabus was a barrier for staff that perceived accommodating further scheduling changes as burdensome.

#### Inner organisational context/structure (schools)

Several themes emerged related to the inner organisational context/structure of schools. Facilitators included teacher’s positive physical activity values and beliefs; executives’ support of PACE and accordingly, teachers’ provision of school day physical activity; and schools with a culture conducive to physical activity that perceived PACE as a useful addition. Barriers included the limited time and competing demands of staff; staff turnover; teacher’s negative physical activity values and beliefs; schools with a culture conducive to physical activity that perceived PACE as unnecessary; and schools without a physical activity culture that did not assign value to it.

**Fig. 4:**
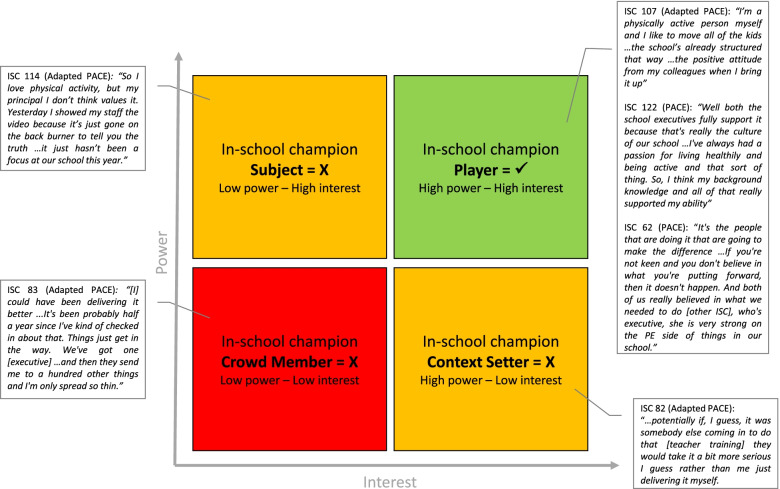
A power-interest matrix that was adapted from Eden & Ackermann, 1998, pg. 349 [43] to show the different categories of in-school champions in the current study with example quote(s) provided for each. Traffic light colours are used to show the likelihood of successful implementation (of strategies) experienced. In-school champion ‘Players’ are shown in green due to their high likelihood of successful implementation. In-school champion ‘Context Setters’ and ‘Subjects’ are shown in yellow due to their potential for successful implementation with further assistance. In-school champion ‘Crowd Members’ are shown in red as they were unlikely to experience successful implementation and faced several challenges

The most prominent inner setting theme was “ISC power-interest” whereby the level of program implementation at each school was proportional to the power and interest of the ISC. This corresponds to stakeholder analysis theory from the organizational strategic management literature, in which stakeholders with high power (ability to influence others due to their position within an organization or devolution) and high interest are considered most salient to achieve objectives. Using pre-defined stakeholder classifications [43], our data showed that ISC ‘Players’ (high power and high interest) were consistently associated with greater implementation of PACE strategies. Conversely, schools with ISC that had low power and/or low interest (‘Subjects’, ‘Context Setters’, and ‘Crowd Members’) were challenged to implement some PACE strategies (Fig. 4).

#### PACE characteristics and processes

The final themes relating to PACE characteristics and processes were implementation facilitators: (i) the adaptable and flexible model providing a variety of physical activity options for teachers; (ii) the observable benefits of the program on students; and (iii) the engaging nature of the intervention via practical hands-on training sessions, expertise and energetic support from project officers, and the provision of quality resources.

### Research objective C: importance of strategies

Table 1 is the joint-display used to address research objective C, with quantitative and qualitative findings juxtaposed in relation to each strategy.

#### Strategy 1: external, ongoing support

This was not considered within the top three for ‘most important strategy’ by any project officer, although the project officer characteristics (e.g., energetic expertise) contributing to an engaging innovation, was a theme for facilitating implementation:

*PACE ISC 62: And we also were lucky enough to have [PO] to come in, and he showed how you could do it easily linked to maths and easily get it within your classrooms for other things as well, which was really good. He was really inspirational. His energy was amazing and the staff really liked having him there … when [PO] came with his energy and made it look so simple, a lot of people instantly started doing it.*

Those schools with ISC ‘Players’ appeared to rely less on this strategy, particularly Adapted PACE ISC that successfully delivered the staff training themselves:

*Adapted PACE ISC 107: … we don't need micro-managing but we also don't need onerous stuff. Every now and then is great and that's fine and email conversation is perfect … so we have a conversation and then we [ISC] keep that alive in our school*.

This strategy had high rates of adoption (all but one school engaged in correspondence with project officers) and reported acceptability by ISC (87%). Qualitative data revealed that limited time and competing demands of staff was a key implementation barrier; suggesting that strategies such as this one which demands little of stakeholders’ time and energy (with responsibility falling on the health service provider) may be highly adopted and acceptable by stakeholders.

Metainferences: This strategy is easily executed and assists with successful program implementation. It is highly important for schools without ISC ‘Players’.

#### Strategy 2: mandate change

This was considered the most important strategy (ranked first) by 5/6 project officers and all interviewed ISC referred to the importance of executive support, except those who were an executive themselves. As such, the presence of a supportive executive within schools was a theme for facilitating implementation:

*PACE ISC 121: … because if you're doing the whole school stuff, then everyone needs to be on board and the best way for this is the executive to lead the way. So whoever's in charge, like one of your deputies or your [assistant principal] are involved and leading it, it's much more likely to be implemented in the classrooms. Yeah, they need to be on board from the start.*

Executive support also equipped ISC with the ‘power’ necessary to successfully implement strategies:

*Project officer 06: The [ISC] who had those executive support or were executives, they were a lot more positive about the program … there was definitely more change in the school and more successful with the entire program.*

Several interviewed project officers and ISC indicated that executives drove the physical activity culture of schools; another theme that facilitated program implementation. In the quantitative findings, 84% of ISC reported that principal support to schedule physical activity was useful/extremely useful. This strategy was also associated with several positive implementation indicators: 100% of schools had a school executive provide verbal commitment to the program during an initial meeting; 80% of surveyed school principals reported a school-level physical activity policy either existing or in the process of being developed; 84% communicated their support for the program to the broader school community; and 72% indicated that PACE was a school priority. However over one quarter (30%) of surveyed teachers did not feel that they had support from their school executive. Further exploration of the qualitative data showed agreeance with these quantitative findings. Whilst executive support was reportedly provided at most schools, there were select schools where it was considerably less prevalent or non-existent:

*Project officer 03: You have some [executives] that'll just go past their desk and they just flick it to, “oh this will go to our sports person” and they don't even look at it again. They don't even come to the staff meeting … We had a mix of schools where that would happen, where we get that kind of high adoption or low adoption.*

Poor executive support was associated with low ISC power, making them a ‘Crowd Member’ or ‘Subject’ and thus hindering their ability to perform responsibilities and implement the program. It was also associated with a school culture that was non-conducive to physical activity in which PACE was insufficient to instigate change. Both of these were identified as key barriers to program implementation.

Metainferences: This strategy is highly important for successful implementation and is also linked to other important strategies. It may be poorly adopted by some schools.

#### Strategy 3: identify and prepare ISC

This was considered an important strategy by all project officers; ranked first by one project officer and within the top three by the other five project officers. This strategy was strongly related to the qualitative theme of ISC power-interest; identifying an ISC ‘Player’ with a high level of power and interest from the onset appeared to be beneficial, if not essential, for program implementation:

*Project Officer 02: It really depends on who the school champion is and who you connected with as to how the school takes it on board … So if the school champion is completely on board and can evoke change and can deliver, get the staff on board.*

The one-day workshop was also considered an important piece contributing to successful program implementation, as it presented an opportunity to enhance ISC interest in the program and power (via self-efficacy) to deliver within their respective school. It also contributed to the engaging innovation via experiential learning and outlined the adaptable/flexible choice-based model – both of which emerged as themes for facilitating implementation. Importantly, the workshop appeared to prepare Adapted PACE ISC to deliver the staff training themselves:

*Adapted PACE ISC 66: Yeah, there was so much in that [workshop] and it was really practical and me, at that time, I wasn't probably an overly sports person at that stage, but I did feel confident that when I left I could teach it because you came away and it was an actual practical workshop.*

Of the surveyed ISC, 78% indicated that having an ISC was useful/extremely useful. From the teacher perspective, 73% indicated that the assistance from their ISC to implement PACE was acceptable. These indicators show that some ISC may not have been engaged in their role. This is consistent with the qualitative findings – although most ISC were ‘Players’, there were select cases with low power and/or interest (‘Context Setters’, ‘Crowd Members’ or ‘Subjects’) which hindered program implementation:

*Adapted PACE ISC 83: [I] could have been delivering it better...It's been probably half a year since I've kind of checked in about that. Things just get in the way. We've got one [executive] … and then they send me to a hundred other things and I'm only spread so thin.*

Metainferences: This strategy is highly important for successful program implementation. It may be poorly implemented by some schools.

#### Strategy 4: develop a formal implementation blueprint

This was not considered one of the top three most important strategies; however project officers noted that it was incorporated into the full day ISC workshop. This strategy contributed to the engaging innovation via quality resources (qualitative theme and sub-theme) that facilitated implementation. A few interviewed project officers also mentioned that such school documents may be used to “handover” information to new staff, thus addressing the barrier of staff turnover (qualitative theme). Of the surveyed stakeholders, 77% of ISC indicated that developing guiding documents such as this was useful/extremely useful and 66% of teachers indicated that it was acceptable in assisting them to schedule physical activity.

Metainferences: This strategy is very easy to execute (included in strategy 3b) and may assist some schools with program implementation.

#### Strategy 5: educational outreach visits

This was considered an important strategy (top three) by 4/6 project officers. It corresponded to several identified implementation facilitators: strategies to enhance teachers’ attitudes, beliefs and level of support; an explanation of the choice-based model; and an engaging innovation via experimental learning to engage staff:

*PACE ISC 62: At first, [staff] were like, "No, there's no way we can fit this in." But then they started realizing that you can incorporate it into other things. And we do it a lot of the time without even thinking about it. So yeah, they were really receptive … I think the staff felt that they had a better grip and handle on what they were doing with the hours.*

Implementation indicators for this strategy were high for the overall sample (> 80% adoption and > 70% acceptability) however compared to PACE schools, Adapted PACE schools had lower adoption (96% vs 71%) and acceptability (81% vs 64%). The use of an ISC to deliver this strategy in Adapted PACE may impede implementation in some schools. Although the majority of ISC felt adequately prepared to deliver this strategy themselves, 18% did not. This is in agreeance with both data sets showing select cases of Adapted PACE ISC that did not proceed to deliver this strategy. This may be due to their limited time/competing demands or leaving the school before delivering the strategy (ISC turnover) – both identified barriers of program implementation. However, qualitative data positioned ISC power-interest as the primary factor that influenced implementation of this strategy at Adapted PACE schools. ISC ‘Players’ successfully delivered this strategy whereas those with low power and low interest struggled or failed to do so:

*Project officer 01: … say in [Adapted PACE] the school champion has been able to go back to the school, organise a time to have the whole school meeting on the agenda, and then provide the presentation [educational outreach] the whole school and teachers have a lot more of an idea … and definitely I think if you've got the support of the executive team. So I know in some of those [Adapted PACE] schools there was an executive that was a school champion, they were able to obviously help get it on the agenda, push it into a staff meeting, organise it.*

PACE used energetic and knowledgeable, external project officers to deliver this strategy which was identified as an implementation facilitator. Regardless, 12% of ISC at PACE schools did not report this strategy as being useful and 18% of teachers did not report it as acceptable. This may be due to several of the identified implementation barriers that are applicable to both intervention groups (PACE and Adapted PACE) such as low executive support, negative attitudes/beliefs of staff, or schools that already prioritised physical activity and felt PACE was unnecessary:

*Adapted PACE ISC 114: I implemented daily fitness prior to even going away to the [ISC workshop] so we're still currently doing that … it hasn't really enriched what we're doing.*

Metainferences: This strategy is important for successful program implementation. ISC ‘Players’ (influenced by strategy 2 and 3) are well positioned to deliver this strategy themselves. Delivery by a project officer may be needed for schools with an ISC ‘Subject’, ‘Context Setter’ or ‘Crowd Member’.

#### Strategies 6, 7 and 8: educational materials, success stories and equipment pack

The resources were considered important (top three) by 2/6 project officers. The quality resources contributing to an engaging innovation emerged as an implementation facilitator, with several interviewed ISC and project officers referring to their usefulness for teachers:

*PACE ISC 122: Yeah, I think the resource is perfect … We can go to them if we ever really need something … it gave us a lot more tools to be able to do it and just physically not having to go and find the resources.*

Quantitative data showed partial implementation for each resource: 65% of surveyed teachers found the online portal useful (43% personally accessed it) and 57% considered the equipment pack acceptable in assisting them to schedule physical activity. The qualitative data expanded on this, showing that the use of resources differed by school depending what they needed and/or found most useful. For example, some schools found the online portal most useful whereas others emphasised the equipment pack and did not use the online portal at all.

Metainferences: The resources are easily executed and may assist schools with program implementation. The importance of each resource varied by school; a range of options permit schools to select and access those that are most useful for them.

### Summary of metainferences

**Fig. 5:**
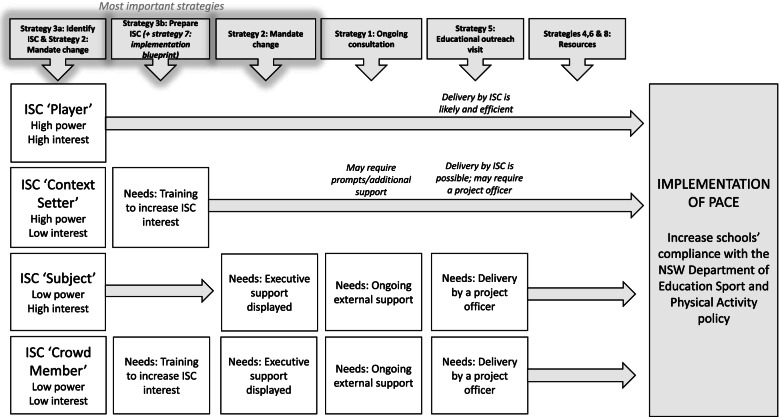
A conceptual model distinguishing pathways for successful implementation based on a synthesis of meta-inferences for strategy importance. Discrete PACE strategies are outlined on the top row with those considered ‘most important’ distinguished. The flow of arrows from left to right shows where there was greater ‘need’ for strategies depending on what category of in-school champion was present at the school. Longer arrows represent less reliance on other strategies

A synthesis of metainferences for the importance of each strategy led to the conceptual model displayed in Fig. 5. No strategies appeared to be discretionary as they differed in adoption, acceptability and usefulness on a school-by-school basis. What worked best and was needed in one school was not necessarily the same for another. However, a few strategies may be considered extremely important as they resulted in improved implementation of the overall program, in addition to remaining strategies. Specifically, nominating an ISC ‘Player’ (via strategy 3a) and reinforcing this by providing them with the power (via strategy 2) and enhanced interest (via strategy 3b) to engage in responsibilities. Schools with an ISC ‘Player’ were more likely to successfully implement PACE whereas schools with an ISC ‘Crowd Member’ faced implementation challenges and required greater support from the other strategies.

## Discussion

Studies exploring, comparing and contrasting implementation strategies for school-based physical activity interventions are rare. We had a novel opportunity to assess the implementation of eight discrete implementation strategies, previously examined through a series of randomised controlled, and noninferiority trials, which collectively improved schools’ compliance with a state-level physical activity policy. The results corroborated the noninferiority trial findings of Adapted PACE being considered “as good as” PACE for increasing teachers’ scheduled minutes of physical activity [24]. The quantitative implementation indicators were generally high for schools in both groups and the barriers and facilitators identified qualitatively were impartial to group allocation. Using state-of-the art mixed methods data integration techniques, we developed meta-inferences for the importance of each discrete implementation strategy and from that, an emergent conceptual model suggesting a path for successful implementation. Although the majority of strategies were important (4/6), two (mandating change and identifying and preparing an ISC) and a related contextual factor (ISC power-interest) emerged as critical to implementation. The findings and related recommendations to optimise PACE are discussed in the context of the literature following.

Our meta-inferences highlighted the importance of the ISC power-interest as a central component to successful implementation. Specifically, we found that schools with an ISC ‘Player’ had higher levels of implementation strategy implementation compared to those with an ISC ‘Crowd Member’. This is supported by stakeholder analysis theory [43] and recommendations to ensure that champions of school-based physical activity programs have the skills, knowledge and disposition to assume the responsibilities expected of them [44]. Further, a 2018 review of champions in healthcare-related implementation identified specific, similar characteristics associated with champions’ effectiveness, including enthusiasm and energy to drive the program, strong educator and presentation skills, having political acumen, a personal belief in the initiative, and being respected and well-liked [45]. Champion attributes may profoundly affect implementation of health interventions, and also the likelihood of their successful scale-up [46, 47]. In the 2007 World Health Organization (WHO) framework for scaling up public health interventions, Simmons and Shiffman [47] state that successful scale-up is more likely with “effective and motivated leaders who command authority and have credibility”. Identifying an ISC ‘Player’ should be a priority of PACE and where this is not possible, every effort should be taken to improve the nominee’s low power and/or interest via the ISC workshop and executive support. Frameworks and measures to identify and classify stakeholders [48, 49] may be a useful starting point for developing guidelines to systematically identify ideal ISC candidates at schools.

Mandating change also emerged as the most important strategy. Specifically, executive support facilitated the implementation of not only the overall program, but also the adoption of other strategies. As the administrative leader within schools, executives have the potential to influence implementation of health programs via endorsement, providing oversight and accountability, and/or enacting formal requirements [50]. In a 2021 evaluation of a school-based participatory health intervention, the level of leadership engagement by school administrators strongly distinguished between high and low implementation schools [51]. Similarly, an evaluation of a multi-strategy school smoking prevention program found that high- and medium- implementation schools had higher levels of administrative leadership than low implementation schools (77.3 and 83.3% vs 42.9%) [52]. The link between executive support and implementation in schools underpins that efforts are needed to address the occasional poor adoption of PACE by some school executives, although this may be naturally addressed under the external policy landscape in NSW (the Department of Education policy and the new PE syllabus). Such “macro-systemic sources of influence” create administrative pressure for schools to implement health programs [50] and may increase executive support of PACE over time. In Canada for example, the AS! BC intervention was rolled out in primary schools in the context of a Provincial Daily Physical Activity policy being implemented. Over three quarters of primary school principals (76%) reported an increased priority assigned to physical activity over a 3 year period due to a combination of AS! BC and the Provincial policy [53].

During intervention design, each PACE strategy was chosen using a robust, theory-based process to overcome identified barriers of the target behaviour [20]. Our findings endorse the final multi-strategy approach, showing that the majority of PACE strategies were important and even those considered less important remained considerably useful for implementation by some schools. This is likely due to the wide variation in factors that influence implementation of physical activity programs in schools [54] including those in the current study. In a series of RCTs undertaken to improve a multi-strategy intervention to assist schools to implement a government nutrition policy, the research team found that removing perceptibly nonessential strategies (to reduce delivery costs) forfeited the intervention effect [55]. From the current findings no PACE strategy appears dispensable; thus those identified as most important should be emphasised in efforts made to improve implementation. It is also important to note that many “less important” strategies were easily implemented within existing infrastructure and represent little opportunity cost to the health service provider and/or schools.

Although there were relatively few implementation barriers to PACE, ‘available time/competing demands of staff’ was prominent, with overt mention in nearly every interview and impacting each of principals, ISC and teachers. Accordingly, PACE strategies with the highest adoption and acceptability were those requiring the least amount of time and effort from targeted stakeholders. In a 2015 systematic review of factors influencing the implementation of school-based physical activity interventions, the most prevalent barrier was time (e.g., competing instructional requirements) [54]. Strategies to assist schools to implement school day physical activity should account for the limited time of staff, although this may be challenging in the context of scale-up which often relies more on organizations for implementation support [47]. This underscores the importance of using ISC ‘Players’, as school staff are more likely to prioritise and deliver a program that they value despite time constraints [54].

This study has several notable limitations. First, stakeholder surveys were finalised prior to our deciding to use a mixed methods approach, which resulted in less congruency between the qualitative and quantitative data collection methods than we would have liked. A prospectively designed mixed methods process evaluation would improve on this in the future. Second, there is a risk of positive response bias from ISC who may have perceived interviewers as associated with PACE delivery personnel, despite clear indication of their separation from the delivery team. Third, although our inductive, data-driven qualitative approach allowed a rich description of the data without constraint to any pre-existing framework [33, 36], future qualitative inquiry in the implementation science field could adopt a more pragmatic approach such as incorporating a framework-driven analysis [56]. Fourth, an essential ingredient within the conceptual model is ISC power-interest. This is based on a comprehensive integration of multiple data sources and rigorous analyses, however a formal comparison of quantitative intervention implementation scores and assignment to a power-interest category for each school is necessary to substantiate our findings. Lastly, although we purposefully sampled for interviews to ensure we had perspectives from a range of schools, we interviewed fewer ISC from the ‘low engagement school’ category due to recruitment challenges. Regardless, we met our total target sample and we were able to triangulate findings across school sites and supplement with project officer’s insight as ‘key informants’ [33, 34], thus achieving sufficient depth and breadth of data for schools of all implementation levels.

## Conclusion

Adapted PACE is the preferred model for delivery at-scale due to its ability to reach a greater proportion of the population at a lower cost to the health service provider, and with an acceptable effect on policy implementation [24]. This study reinforces this and shows that identification and selection of ISC who are ‘Players’ and demonstration of executive support are very important (if not essential) for successful policy implementation and consequential impact. These findings expose opportune ways to optimise PACE with minimal adaptations and at no additional cost to the health service provider. Given these strategies are commonly employed to improve the implementation of policies and practices in the school setting [57], our findings may contribute to improving implementation of school health interventions broadly.

## Supplementary Information

**Additional file 1.**

**Additional file 2:** **Table S3.** Results from thematic analysis - Main influences of implementation with example quotes from interviews of in-school champions and project officers.

## Data Availability

The datasets used for the current study are available from the corresponding author upon reasonable request.

## Abbreviations

PACE: Physically Active Children in Education
TDF: Theoretical Domain Framework (TDF)
BCW: Behaviour Change Wheel
RCT: Randomised controlled trial
MRC: Medical Research Council
NSW: New South Wales
ISC: In-school champion
PE: Physical Education
PDHPE: Personal Development, Health and Physical Education
NESA: NSW Education Standards Authority
WHO: World Health Organization

## References

[1] Linnan L, Steckler A (2002). Process evaluation for public health interventions and research.

[2] Craig P, Dieppe P, Macintyre S, Michie S, Nazareth I, Petticrew M (2008). Developing and evaluating complex interventions: the new medical research council guidance. BMJ.

[3] Moore GF, Audrey S, Barker M, Bond L, Bonell C, Hardeman W (2015). Process evaluation of complex interventions: medical research council guidance. BMJ.

[4] Glasgow RE, Vogt TM, Boles SM (1999). Evaluating the public health impact of health promotion interventions: the RE-AIM framework. Am J Public Health.

[5] van de Glind I, Bunn C, Gray C, Hunt K, Andersen E, Jelsma J (2017). The intervention process in the European fans in training (EuroFIT) trial: a mixed method protocol for evaluation. Trials.

[6] Powell BJ, Fernandez ME, Williams NJ, Aarons GA, Beidas RS, Lewis CC (2019). Enhancing the impact of implementation strategies in healthcare: a research agenda. Front Public Health.

[7] Powell BJ, Beidas RS, Lewis CC, Aarons GA, McMillen JC, Proctor EK (2017). Methods to improve the selection and tailoring of implementation strategies. J Behav Health Serv Res.

[8] Powell BJ, Waltz TJ, Chinman MJ, Damschroder LJ, Smith JL, Matthieu MM (2015). A refined compilation of implementation strategies: results from the expert recommendations for implementing change (ERIC) project. Implement Sci.

[9] Auditor-General NSW (2012). Physical activity in government primary schools. In: Department of Education and Communities, editor.

[10] Olstad DL, Campbell EJ, Raine KD, Nykiforuk CI (2015). A multiple case history and systematic review of adoption, diffusion, implementation and impact of provincial daily physical activity policies in Canadian schools. BMC Public Health.

[11] Canadian Fitness and lifestyle Research Institute (CFLRI) (2016). School policies supporting physical activity and sport.

[12] Oxford Research. Bevægelse i skoledagen: Populationsundersøgelse 2017 Udarbejdet af Oxford Research for Dansk Skoleidræt og TrygFonden (in Danish). Denmark: Oxford Research; 2017. Available from: https://skoleidraet.dk/media/6346522/bevaegelse-i-skoledagen-2017.pdf.

[13] Carlson JA, Sallis JF, Chriqui JF, Schneider L, McDermid LC, Agron P (2013). State policies about physical activity minutes in physical education or during school. J Sch Health.

[14] Harrington DM, Belton S, Coppinger T, Cullen M, Donnelly A, Dowd K (2014). Results from Ireland’s 2014 report card on physical activity in children and youth. J Phys Act Health.

[15] Hardman K (2008). Physical education in schools: a global perspective. Kinesiol Int J Fundam Appl Kinesiol.

[16] Weatherson KA, Gainforth HL, Jung ME (2017). A theoretical analysis of the barriers and facilitators to the implementation of school-based physical activity policies in Canada: a mixed methods scoping review. Implement Sci.

[17] Nathan N, Hall A, McCarthy N, Sutherland R, Wiggers J, Bauman AE (2021). Multi-strategy intervention increases school implementation and maintenance of a mandatory physical activity policy: outcomes of a cluster randomised controlled trial. Br J Sports Med..

[18] Mâsse LC, Naiman D, Naylor P-J (2013). From policy to practice: implementation of physical activity and food policies in schools. Int J Behav Nutr Phys Act.

[19] Gilmore T, Donohoe H (2016). Elementary school generalist teachers’ perceived competence to deliver Ontario’s daily physical activity program. Loisir et Soc /Soc Leis.

[20] Nathan N, Wiggers J, Bauman AE, Rissel C, Searles A, Reeves P (2019). A cluster randomised controlled trial of an intervention to increase the implementation of school physical activity policies and guidelines: study protocol for the physically active children in education (PACE) study. BMC Public Health.

[21] Nathan N, Elton B, Babic M, McCarthy N, Sutherland R, Presseau J (2018). Barriers and facilitators to the implementation of physical activity policies in schools: a systematic review. Prev Med.

[22] New South Wales (NSW) Government (2015). Sport and physical activity policy. Department of Education.

[23] Nicole KN, Rachel LS, Kirsty H, Nicole JM, Matthew P, Ben E (2020). Implementation of a school physical activity policy improves student physical activity levels: outcomes of a cluster-randomized controlled trial. J Phys Act Health.

[24] IN REVIEW. L, C, Wolfenden L, Hall A, Sutherland R, Naylor PJ, Oldmeadow C, et al. Optimising a multi-strategy implementation intervention to improve the delivery of a school physical activity policy at scale: Findings from a randomised noninferiority trial. Int J Behav Nutr Phys Act. 2021. 10.1186/s12966-022-01345-6 PMC9392334 35987776

[25] Cook CR, Lyon AR, Locke J, Waltz T, Powell BJ (2019). Adapting a compilation of implementation strategies to advance school-based implementation Research and practice. Prev Sci.

[26] McCrabb S, Mooney K, Elton B, Grady A, Yoong SL, Wolfenden L (2020). How to optimise public health interventions: a scoping review of guidance from optimisation process frameworks. BMC Public Health.

[27] Wolfenden L, Bolsewicz K, Grady A, McCrabb S, Kingsland M, Wiggers J, et al. Optimisation: defining and exploring a concept to enhance the impact of public health initiatives. Health Res Policy Syst. 2019;17(1). 10.1186/s12961-019-0502-6 PMC6937822 31888666

[28] Education NSW (2021). 2020 NSW government schools by type and SA4 groupings.

[29] McKay H, Naylor P-J, Lau E, Gray SM, Wolfenden L, Milat A (2019). Implementation and scale-up of physical activity and behavioural nutrition interventions: an evaluation roadmap. Int J Behav Nutr Phys Act..

[30] Creswell JW. A concise introduction to mixed methods research. Thousand Oaks: Sage Publications; 2015.

[31] Padgett DK (2012). Qualitative and mixed methods in public health.

[32] Flight L, Julious SA (2016). Practical guide to sample size calculations: non-inferiority and equivalence trials. Pharm Stat.

[33] Patton MQ (2015). Qualitative research & evaluation methods.

[34] Hamilton AB, Finley EP (2019). Qualitative methods in implementation research: an introduction. Psychiatry Res.

[35] QSR International Pty Ltd. NVivo. Available from: https://www.qsrinternational.com/nvivo-qualitative-data-analysis-software/home. (released Mar 2020).

[36] Braun V, Clarke V (2006). Using thematic analysis in psychology. Qual Res Psychol.

[37] Hill CE, Knox S, Thompson BJ, Williams EN, Hess SA, Ladany N (2005). Consensual qualitative research: an update. J Couns Psychol.

[38] O’Cathain A, Murphy E, Nicholl J (2010). Three techniques for integrating data in mixed methods studies. BMJ.

[39] Guetterman TC, Fetters MD, Creswell JW (2015). Integrating quantitative and qualitative results in health science mixed methods research through joint displays. Ann Fam Med.

[40] Tashakkori A, Teddlie C (2008). Quality of inferences in mixed methods research: calling for an integrative framework. Adv Mixed Methods Res: Sage Publication.

[41] Damschroder LJ, Aron DC, Keith RE, Kirsh SR, Alexander JA, Lowery JC (2009). Fostering implementation of health services research findings into practice: a consolidated framework for advancing implementation science. Implement Sci.

[42] NSW Education Standards Authority (NESA) (2018). Personal development, health and physical education (PDHPE) K–10 syllabus.

[43] Eden C, Ackermann F (1998). Making strategy: the journey of strategic management.

[44] Carson RL, Castelli DM, Pulling Kuhn AC, Moore JB, Beets MW, Beighle A (2014). Impact of trained champions of comprehensive school physical activity programs on school physical activity offerings, youth physical activity and sedentary behaviors. Prev Med.

[45] Miech EJ, Rattray NA, Flanagan ME, Damschroder L, Schmid AA, Damush TM (2018). Inside help: an integrative review of champions in healthcare-related implementation. Sage Open Med.

[46] Milat AJ, Bauman A, Redman S (2015). Narrative review of models and success factors for scaling up public health interventions. Implement Sci.

[47] Simmons R, Shiffman J (2007). Scaling up health service innovations: a framework for action. Scaling up health service delivery: from pilot innovations to policies and programmes.

[48] Yang J, Shen GQ, Bourne L, Ho CMF, Xue X (2011). A typology of operational approaches for stakeholder analysis and engagement. Constr Manag Econ.

[49] Schiller C, Winters M, Hanson HM, Ashe MC (2013). A framework for stakeholder identification in concept mapping and health research: a novel process and its application to older adult mobility and the built environment. BMC Public Health.

[50] Domitrovich CE, Bradshaw CP, Poduska JM, Hoagwood K, Buckley JA, Olin S (2008). Maximizing the implementation quality of evidence-based preventive interventions in schools: a conceptual framework. Adv Sch Ment Health Promot.

[51] Wilhelm AK, Schwedhelm M, Bigelow M, Bates N, Hang M, Ortega L (2021). Evaluation of a school-based participatory intervention to improve school environments using the consolidated framework for implementation Research. BMC Public Health.

[52] Bast LS, Due P, Ersbøll AK, Damsgaard MT, Andersen A (2017). Association of School Characteristics and Implementation in the X: IT study—a school-randomized smoking prevention program. J Sch Health.

[53] McKay HA, Macdonald HM, Nettlefold L, Masse LC, Day M, Naylor P-J (2015). Action schools! BC implementation: from efficacy to effectiveness to scale-up. Br J Sports Med.

[54] Naylor P-J, Nettlefold L, Race D, Hoy C, Ashe MC, Higgins JW (2015). Implementation of school based physical activity interventions: a systematic review. Prev Med.

[55] Reilly KL, Reeves P, Deeming S, Yoong SL, Wolfenden L, Nathan N (2018). Economic analysis of three interventions of different intensity in improving school implementation of a government healthy canteen policy in Australia: costs, incremental and relative cost effectiveness. BMC Public Health.

[56] Ramanadhan S, Revette AC, Lee RM, Aveling EL (2021). Pragmatic approaches to analyzing qualitative data for implementation science: an introduction. Implement Sci Commun.

[57] Wolfenden L, Nathan NK, Sutherland R, Yoong SL, Hodder RK, Wyse RJ, et al. Strategies for enhancing the implementation of school-based policies or practices targeting risk factors for chronic disease. Cochrane Database Syst Rev. 2017;11:CD011677. 10.1002/14651858.CD011677.pub2 PMC6486103 29185627

