# Supplementary material for: Identifying essential implementation strategies: a mixed methods process evaluation of a multi-strategy policy implementation intervention for schools
Source: Int J Behav Nutr Phys Act. 2022 Apr 12;19:44. doi: 10.1186/s12966-022-01281-5 (PMC9004180; doi:10.1186/s12966-022-01281-5)
Supplement: Supplementary file 1 — Additional file 1. [file 12966_2022_1281_MOESM1_ESM.docx]

Additional File 1: Quantitative data collection tools and respective measures used to assess implementation indicators (as defined by McKay et al***;** Research objective A) and perceived usefulness (Research objective C) for each strategy

| **Strategies** | **Dose delivered**  ***Intended units delivered*** | **Fidelity**  ***The extent to which strategies were implemented as prescribed*** | **Adoption**  ***Proportion and representativeness of school stakeholders that utilized strategies*** | **Acceptability**  ***Perceptions among school stakeholders that strategies were agreeable, palatable or satisfactory*** | **Usefulness**  ***Perceptions among school stakeholders that strategies were useful for program implementation*** |
| --- | --- | --- | --- | --- | --- |
| 1. External, ongoing support | **Tool**: project records  **Measure**: Records of emails/phone-call/ in-person visits between ISC and project officers (at-least three with pre-defined criteria at intervention onset, mid-way and follow-up) | **Tool**: project records  **Measure**: The number of schools with an ISC that engaged in the communication with project officers (at least 2 occurrences of communication) to carry out tasks expected of them | **Tool**: project records  **Measure**: The number of schools in which an ISC engaged in communication with project officers (at least 2 occurrences of in-person, email or telephone communication recorded) | **Tool**: ISC survey  **Measure**: The support I received from the Good for Kids project officer to implement the Good for Kids physical activity program at my school was adequate. (1=strongly disagree, 2=disagree, 3=neither agree nor disagree, 4=agree, 5=strongly agree)  *Adapted PACE included the additional measure: Did you feel adequately prepared to deliver the whole school one-hour face to face professional learning session without a Good for Kids project officer? (yes/no/unsure) | N/A |
| 2. Mandate change   1. Principal meeting | **Tool**: project records  **Measure**: For each school, records of email/phone-call/ in-person visit between ISC and principal and checklist with pre-defined agenda items (i.e., what PACE entails; principal responsibilities; ISC nomination and responsibilities; and verbal consent) | **Tool**: project records  **Measure**: The number of schools with principals that provided verbal commitment to undertake program and responsibilities expected of them. | **Tool**: project records  **Measure**: The number of schools that had a school executive meet with a project officer. | N/A | **Tool**: ISC survey  **Measure:** Please indicate your perception of how useful each resource was: having principal/ executive support to schedule physical activity. (1=not useful at all, 2=not useful, 3=neither useful nor not useful, 4=useful, 5=extremely useful) |
| 1. School policy |  |  | **Tool**: principal survey  **Measure**: Which of the following statements best represents your school’s intent to implement a physical activity policy? (we have not thought about it; we are thinking about it; we are planning to take some steps/in development; we currently have a school physical activity policy) | N/A |  |
| 1. Principal display support |  |  | **Tool**: principal survey  **Measures**:  -Whilst this program was running in your school were you able to communicate your support for the implementation of the physical activity policy / guideline through announcements to teachers for example in staff meetings? (yes/no)  -Is the PACE program regarded as a priority area in your school? (yes/no/don’t know)  **Tool**: teacher survey  **Measure**: I have support from my school executive to implement the Good for Kids school physical activity program. (1=strongly disagree, 2=disagree, 3=neither agree nor disagree, 4=agree, 5=strongly agree) | N/A |  |
| 3. Identify and prepare champions   1. ISC within school | **Tool**: project records  **Measure**: Records of the ISC workshops facilitated by project officers; completed fidelity checklist for each. | **Tool**: project records  **Measure**: The number of ISC that attended a workshop and actively participated in activities | **Tool**: teacher survey  **Measure**: I have support from my in-school champion to implement the Good for Kids school physical activity program. (1=strongly disagree, 2=disagree, 3=neither agree nor disagree, 4=agree, 5=strongly agree) | **Tool**: teacher survey  **Measure**: The assistance I received from my in-school champion was acceptable in assisting me to schedule physical activity in my class. (1=strongly disagree, 2=disagree, 3=neither agree nor disagree, 4=agree, 5=strongly agree) | **Tool**: ISC survey  **Measure**: Please indicate your perception of how useful each resource was: having an ISC located within the school (1=not useful at all, 2=not useful, 3=neither useful nor not useful, 4=useful, 5=extremely useful) |
| 1. ISC workshop |  |  | **Tool**: project records  **Measure**: The number of ISC that attended a workshop. | N/A |  |
| 4. Develop a formal implementation blueprint |  | *As per strategy 3* | **Tool**: project records  **Measure**: The number of ISC that actively participated in a workshop (this took place during strategy 3b) | **Tool**: teacher survey  **Measure**: The school physical activity plan developed by my in-school champion was acceptable in assisting me to schedule physical activity in my class. (1=strongly disagree, 2=disagree, 3=neither agree nor disagree, 4=agree, 5=strongly agree) | **Tool**: ISC survey  **Measure**: Please indicate your perception of how useful each resource was: the creation of the school physical activity policy, scope and sequence document and whole-school physical activity timetable. (1=not useful at all, 2=not useful, 3=neither useful nor not useful, 4=useful, 5=extremely useful) |
| 5. Conduct educational outreach visits | **Tool**: project records  **Measure**: For PACE schools, records of staff training sessions facilitated by project officers, inclusive of educational PowerPoint presentation and interactive games/activities. For Adapted PACE schools, email or telephone confirmation from ISC that this had been delivered per protocol. |  | **Tool**: project records  **Measure**: The number of schools that accepted and participated in the staff training session. | **Tool**: teacher survey  **Measures**:  Overall- The whole school staff meeting was acceptable in assisting me to schedule physical activity in my class. (1=strongly disagree, 2=disagree, 3=neither agree nor disagree, 4=agree, 5=strongly agree)  Content- The information provided at the whole school meeting was acceptable in assisting me to schedule physical activity in my class. (1=strongly disagree, 2=disagree, 3=neither agree nor disagree, 4=agree, 5=strongly agree)  **Tool**: ISC survey (Adapted PACE only)  **Measure**: Did you feel adequately prepared to deliver the whole school one-hour face to face professional learning session without a Good for Kids project officer? (yes/no/unsure) | **Tool**: ISC survey (PACE only)  **Measure**: Please indicate your perception of how useful each resource was: Having the project officer conduct the one-hour face to face professional development meeting for all teachers. (1=not useful at all, 2=not useful, 3=neither useful nor not useful, 4=useful, 5=extremely useful) |
| 6. Develop and distribute educational materials   1. ISC manual | **Tool**: project records  **Measure**: For each school, a checklist with the date the resource was distributed to schools/school stakeholders. | **Tool**: project records  **Measure**: The number of schools with an ISC that received the manual; accessed the online portal; and viewed professional learning videos | **Tool**: project records  **Measure**: The number of ISC that attended a workshop (this was distributed during strategy 3b) | N/A | **Tool**: Teacher survey  **Measure**: Did you find the resources available through the online portal useful in helping you to schedule physical activity? (yes/no) |
| 1. Educational materials in-print and online portal |  |  | **Tool**: teacher survey  **Measures**:  Resources- Have you used the Good for Kids resources e.g., online portal, equipment pack, FMS booklet? (yes/no)  Online portal- Have you accessed the resources on the online portal? (yes/no)  **Tool**: project records (web analytics)  **Measure**: The number of schools that had at least one teacher log in to their online portal account. | **Tool**: teacher survey  **Measure**: The information on the online portal was acceptable in assisting me to schedule physical activity in my class. (1=strongly disagree, 2=disagree, 3=neither agree nor disagree, 4=agree, 5=strongly agree) |  |
| 1. Professional learning videos on online portal |  |  | **Tool**: project records (web analytics)  **Measure**: The number of schools that had one or more staff view at least one of the online learning videos. |  |  |
| 7. Capture and share local knowledge | **Tool**: project records  **Measure**: As above. | *As per strategy 6 (access the online portal)* | **Tool**: project records (web analytics)  **Measure**: The number of schools that had at least one teacher log in to their online portal account. | **Tool**: teacher survey  **Measure**: (Repeat) The information on the online portal was acceptable in assisting me to schedule physical activity in my class. (1=strongly disagree, 2=disagree, 3=neither agree nor disagree, 4=agree, 5=strongly agree) | N/A |
| 8. Change physical structure and equipment   1. Equipment pack provided | **Tool**: project records  **Measure**: As above. | **Tool**: project records  **Measure**: The number of schools that received an equipment pack (*as per strategy 3*); and developed further equipment packs for their classroom. | **Tool**: project records  **Measure**: The number of schools that received an equipment pack (this was distributed during strategy 3b)  **Tool**: teacher survey  **Measures**: (Repeat) Have you used the Good for Kids resources e.g., online portal, equipment pack, FMS booklet? (yes/no) | **Tool**: teacher survey  **Measure**: The equipment pack provided from the Good for Kids team was acceptable in assisting me to schedule physical activity in my class. (1=strongly disagree, 2=disagree, 3=neither agree nor disagree, 4=agree, 5=strongly agree) | N/A |
| 1. Schools develop additional packs |  |  | **Tool**: ISC survey  **Measure**: Please mark one choice if you conducted the following activity: Purchase equipment packs for all classrooms. (yes/partially/no) | N/A |  |
| *ISC: In-school champion*  **McKay H, Naylor P-J, Lau E, Gray SM, Wolfenden L, Milat A, et al. Implementation and scale-up of physical activity and behavioural nutrition interventions: an evaluation roadmap. International Journal of Behavioral Nutrition and Physical Activity. 2019;16(1).* | | | | | |
