# Supplementary material for: Identifying essential implementation strategies: a mixed methods process evaluation of a multi-strategy policy implementation intervention for schools
Source: Int J Behav Nutr Phys Act. 2022 Apr 12;19:44. doi: 10.1186/s12966-022-01281-5 (PMC9004180; doi:10.1186/s12966-022-01281-5)
Supplement: Supplementary file 2 — Additional file 2: Table S3. Results from thematic analysis - Main influences of implementation with example quotes from interviews of in-school champions and project officers. [file 12966_2022_1281_MOESM2_ESM.docx]

Table 3: Results from thematic analysis - Main influences of implementation with example quotes from interviews of in-school champions and project officers

| Category | Themes and sub-themes | Sample quotes |
| --- | --- | --- |
| External policy landscape  (External policies that exerted pressure on schools to participate in PACE [e.g., state or district policies]) | Department of Education Sport and Physical Activity policy  *The government mandate requires that NSW teachers schedule 150 minutes of weekly physical activity; teachers must schedule the physical activity that is promoted and supported by PACE. The mandatory nature of this policy facilitated the implementation of PACE.* | ISC 62 (P): And it was our attitude as well with them, saying to them, "This is really important. It's a focus all over Australia for healthy kids. This just isn't something we do, this is part of their learning and it's mandatory. These hours are mandatory. So, work out how you can fit it in"…So people are now knowing that this must be programmed, this is something that is not just an extra, this is part of our hours for PE and it has to be registered.  ISC 110 (P): And I think initially they thought the 150 minutes was me, just made up a number and was like, "This is what you need to do". So once they were aware …once we kind of rolled out that and showed them the stats [policy requirement] everyone was more than happy.  ISC 82 (AP): …so all the classes, all the teachers had access to a OneNote file that we could go onto and log our activity for each day which highlighted that we were definitely not meeting the required 150 minutes of physical activity a week. So then we got together and came up with some ways that we could improve that.  ISC 83 (AP): We didn't want to overload the teachers, and a lot of the stuff they've been doing in their classrooms regardless …but just highlighting that it is important and …it is mandatory that we do get those minutes as well. I think that was the most important thing that we got back.  PO 01: …if we've got some buy-in from the directors or regional directors saying that, "You need to have these policies. They are mandated policies."…It is a mandatory component of their day-to-day school, it is a mandatory key learning area of 150 minutes. |
|  | New PE syllabus  **-**  *In 2018 the NSW Education Standards Authority (NESA) released a new Personal Development, Health and Physical Education (PDHPE) syllabus requiring primary school students to participate in 1.5 - 2.5 hours per week. For some schools accommodating for PACE was seen as burdensome in addition to the new syllabus [-]. For other schools, the new syllabus provided an opportunity to revamp and focus on scheduling physical activity, including that promoted and supported by PACE [+].* | ISC 110 (P): [+] The fact that this new PE syllabus comes out, it's already out, but it's mandatory to be taught from 2020 – that's really helped as well. So it's kind of been that clean slate ...  ISC 62 (AP): [-] It was very hard going with the new [PE] syllabus, but that was probably the biggest thing we found was a problem …they felt that they'd already put enough change and enough on their plate with the new syllabus and all these changes …Because it is such a different syllabus, compared to what we had ...That in itself was enormous and so some of the staff couldn't do that and the next step …  PO 05: [+] But that older generation of teaching, it's a little bit harder because they're sort of stuck in their ways. They like to do this, this way. But with the new curriculum that's come out, I think it's shaken up a few things and there's a couple of opportunities there.  PO 06: [+] And also the enablers of having scope and sequences linked to the new [PE] syllabus was definitely an enabler too, because many schools are only just transitioning to the new syllabus. So offering those new ways of using the syllabus was definitely an enabler. |
| Inner organisational context/structure  (School culture, champions, level of support and staff or resource availability/turnover) | Teachers' attitudes, beliefs and level of support  **-**  *Teachers' perspectives of physical activity were varied. Those teachers who valued school day physical activity were more likely to engage in the PACE program and its intended actions (scheduling school day physical activity) [+] compared with those teachers who did not perceive school day physical activity as important and/or lacked confidence [-].* | ISC 122 (P): [+] …we're all sort of like-minded in that we're all active people anyway and very interested in that area. So, it sort of came as sort of second nature, I guess to our school.  ISC 110 (P): [+ -] I'd say 80% [teachers] were more than happy to jump on board. And there was a few there that were a bit, they just value different things. So in their classrooms they're really heavily technology-based and they were kind of like, "How are we gonna fit in the time for them to run around?"  ISC 107 (AP): [+] …just the positive attitude from my colleagues where when I bring it up, they're not saying "oh here we go again another burden, another layer of hard work", so it's a positive response from them so it doesn't become a burden.  ISC 114 (AP): [-] I think a lot of staff, they don't like teaching PE. It's one of those things that they hate doing, they just think, "I'll leave it to the young teachers, they'll do it."  ISC 66 (AP): [-] I think at first, most were probably saying, "Oh, this is more work." It could be a bit daunting if they weren't athletic or naturally sporty themselves … not everybody likes change. And at first, people were very resistant to the change.  PO 05: [+ -] And then also probably the teacher's interest as well. If they're not interested in it themselves, they're not going to pursue it either …you find a lot of them are just, they're teaching the same way because that's what they're comfortable in doing and that's what they like to do and it's how they've always done it and how they've been trained.  PO 06: [-] And also just personal belief systems of teachers about physical activity. Some found that it would have been very difficult for them to actually teach those lessons themselves personally, and I think that may have been a barrier to whole school implementation. |
|  | Supportive executive  *Executives that supported PACE and accordingly, teacher's provision of school day physical activity, were considered extremely useful (and in some cases, essential) for successful program implementation.* | ISC 76 (P): [The principal is] actually on board …even as we're writing the programs now, they're being involved and they're instrumental in guiding those people who don't timetable enough sports to work out the hours, make sure that if they don't have the skill to buddy up with a class that has got good PE program  ISC 110 (P): Yeah, all the executives have been really good. When I did the resourcing one of our APs [assistant principal] was excellent; we went through, we ordered what we needed and kind of connected it to the sporting schools program that we run in the school ...that's been really awesome and cost effective.  ISC 121 (P): …because if you're doing the whole school stuff, then everyone needs to be on board and the best way for this is the executive to lead the way, so whoever's in charge, like one of your deputies or your APs are involved and leading it, it's much more likely to be implemented in the classrooms. Yeah, they need to be on board from the start. And participate is the other thing …once they see that even the principal's down there doing it, it becomes a second nature to people in the school and it's just expected that they do it.  ISC 66 (AP): It [executive support] is so important. Particularly because with our curriculum there's a huge focus on wellbeing and that should be at the forefront of our teaching and teaching pedagogy.  PO 02: …the more the principal or exec were on board and present, then the better their [school] implementation was.  PO 03: I think initially that has to come from the top. I think that's the huge thing to get them to schedule it …if someone in the school isn't leading that or driving it or coming from the top or the principal's not checking on it or the execs aren't checking in then ...those that don't see it a priority won't. So I think that that's the initial one. I'd probably say that's first and foremost.  PO 04: Well, number one would be the top down support from the principal …the priorities really come from the principal and then flow down to the staff depending on what direction they want to take. So I'd say that's one of the main ones, because if the principal's not on-board, seen to be active in promoting it and supporting it, then teachers will pick that up straight away and probably not get on board as much as they should. |
|  | Staff turnover  **-**  *Loss of knowledge during staff transition posed a challenge for implementing the program. This may also impede program sustainability regardless of school’s initial implementation levels.* | ISC 107 (AP): We're about to change our head of junior school, our current ones are moving away; we're getting a new one next year. We don't know what the leadership angle is gonna be. So there's a sense of unknown.  ISC 66 (AP): …just the handover of information that it's all there and not taken with the teacher who leaves …if that teacher leaves and they're the only one trained in it then it just doesn't happen. I think they probably need to allocate and make sure a person, irrespective ...probably just the paperwork on their end too, is kept in a file where it's all accessible.  ISC 114 (AP): Yeah, so I won't be here at this school next year, I'm moving to another school. So I guess, you know, how does physical activity continue in our school? There's I guess a bit of a teacher turnover here. So you start the program in the first year but then maybe we need to have a sustaining year in that following year.  PO 01: And we're finding, from a sustainability point of view; during the school year, at the end of the school year, into a new school year, you'll find a change in staff …if you don't have that shifting culture and you've got new staff coming in, they won't know that it is a big part of the school that they've come to – that physical activity is a key learning area.  PO 03: it's a little bit of turnover I think that…You know with school champions moving on …being able to contact schools was difficult at times; change of staff, and the time frame… |
|  | Limited time/competing demands of staff  **-**  *Some schools found that the busy schedules of staff (principals or teachers) made it difficult to implement PACE strategies that required their time.* | ISC 76 (P): As I said to you, we were late in getting back to [PO] and we are negligent in that regard. But I think it's just we [teachers] are time poor.  ISC 110 (P): It's hard; they [school executives] are time poor. So when you're coming to them like, "Can you look at this?" or when I was writing the policy, I think that a little bit more time could have been given.  ISC 82 (AP): Everything just gets so busy, and things start piling on top of each other. It happens.  PO01: Probably the biggest barrier is still time. It seems to come up regularly with those teachers and schools that I've spoken to …I guess the competing demands of the other key learning areas that they have every day in the classroom.  PO 02: They [ISC] are usually really on board and want their staff to do it, but it's time that usually is the only thing they whinge about, like they just haven't had time to get stuff done or haven't had the chance to get their staff to do what they need them to do.  PO 03: And then just getting a time with the schools to run the staff meeting, for them to reply to emails, and that kind of stuff, to meet with the school champions, like all that kind of stuff. Time has probably been the biggest barrier. |
|  | School physical activity culture  **-**  *The value/importance that schools assigned to physical activity ranged from low to high amidst varied levels of implementation. In schools that already valued and prioritised physical activity, PACE was perceived as either a useful addition [+] or unnecessary [-]. In those schools that did not appear to value physical activity, PACE was insufficient to instigate change [-].* | ISC 122 (P): [+] So, it sort of came as second nature, I guess to our school ... it's what we sort of do anyway with our outdoor education, and the sort of culture that we have at our school already. So, it was really good because it supplemented that, but that sort of happened naturally for us.  ISC 107 (AP): [+] So it's already part of our culture, its already part of our planning, it's already part of our thinking. So as I've said before, all that was in place. It was more of a case of tweaking the timetable to ensure that the movement of the children was planned and not let slide away.  ISC 114 (AP): [-] I implemented daily fitness prior to even going away to the [ISC workshop] so we're still currently doing that. We also have our PE lessons plus we also have our sport day as well on Friday …so yeah, it hasn't really enriched what we're doing …it's just gone on the back burner to tell you the truth …it just hasn't really been a priority here at our school.  PO 05: [-] It's got to be a priority within the school. So, if it isn't a priority with them, they're not going to really push for it, they're just going to keep doing their same everyday thing …it really is a case-by-case basis. |
|  | ISC power-interest  **-** | |
|  | 'Player': high power-high interest  *ISC with high power and high interest were more likely to have successful program implementation at their school. The majority of interviewed ISC fell into this category. High power resulted from (i) direct support from a school executive, (ii) being in an executive role themselves, or (iii) having an established position of influence within the school. High interest was the result of (i) a personal interest in physical activity, (ii) valuing school day physical activity for students and/or (iii) prioritising the program responsibilities bestowed to them.* | ISC 122 (P): Well both the school executives fully support it because that's really the culture of our school …I've always had a passion for living healthily and being active and that sort of thing. So, I think my background knowledge and all of that really supported my ability …I sort of came initial well-armed.  ISC 62 (P): it's the people that are doing it that are going to make the difference …If you're not keen and you don't believe in what you're putting forward, then it doesn't happen. And both of us really believed in what we needed to do, [other ISC] who's executive, she is very strong on the PE side of things in our school so that is her area. I PE trained 30 years ago, so I have a belief in that area, but I think that it all depends on the person. It's like anything, your leader is the one that starts.  PO 01: …you need the executive buy-in to help that in-school champion disseminate and deliver it. If it's only one classroom teacher trying to change the whole school, that's going to be pretty challenging …So I know in some of those [Adapted PACE] schools there was an executive that was a school champion, they were able to obviously help get it on the agenda, push it into a staff meeting, organize it.  PO 02: It really depends on who the school champion is and who you connected with as to how the school takes it on board. So originally I feel that it's very positive when we start with them, and then it's what happens next is the varied part. So whether they take back from the training and do anything is the important part …I think it really depends on the school champion. So if the school champion is completely on board and can evoke change and can deliver, get the staff on board, then you don't need it [PACE: staff training delivered by PO].  PO 03: It needs to be that right person who just has that respect from the staff and can take the time to make sure that they're promoting it in the school …It's not just a teacher signing up to get a few hours, they [ISC] actually need to be able to lead this in the school. It needs to be the right staff member who has that initiative to take it on … every school can be so different …the school champion either being an exec, which isn't always possible, or just having the support from them is really important.  PO 04: In my schools, I'd say 70-75% felt the program was very positive. And I'd equate that to the fact that the school champion was quite interested in the program and then passed on that enthusiasm to the staff. Really, it depends on the school champion and the principal.  PO 06: It [implementation] was varied. Depending on who the ISC was, and what their role was in the school …The ones who had those executive support or were executives, they were a lot more positive about the program; there was definitely more change in the school and more successful with the entire program …The ones that were just the teacher champions, they had a lot of problems implementing. |
|  | 'Subject': low power-high interest  **-**  *ISC with low power and high interest had challenges in implementing the program at their school. Low power resulted from being a new teacher and/or minimal executive support.* | ISC 114 (AP): So I love physical activity, but my principal I don't think values it. yesterday I showed my staff the video because it's just gone on the back burner to tell you the truth …it just hasn't been a focus at our school this year…I think that what would be really beneficial is like one: so the professional development I got – being that full-on, hands-on, activity-based stuff – that if someone from Good for Kids actually came out to our school and provided that to our staff. Because I find that, in everything, teachers respond better when someone from the outside come in because they're the expert in it.  PO 06: I dealt with a couple of champions who were first year out teachers and were given the opportunity to go to the workshops, but then found it extremely difficult to implement in their school, because they were just new teachers. |
|  | 'Context Setter': high power-low interest  **-**  *ISC with high power and low interest were less motivated to implement the program in their school, hindering implementation [-].* | ISC 82 (AP): Our new principal, yes, very supportive…potentially if, I guess, it was somebody else coming in to do that [teacher training] they would take it a bit more serious I guess rather than me just delivering it myself. Just with the start of the year and I think we had a lot of other physical literacy already booked in and so by the time that we did get around to doing that it was quite late. I think it probably would have been more impactful if we’d done it earlier. |
|  | 'Crowd Member': low  **-**  Power-low interest  *ISC with low power and low interest were less successful implementing the program at their school.* | ISC 83 (AP): [I] could have been delivering it better ...It's been probably half a year since I've kind of checked in about that. Things just get in the way. We've got one [executive] …and then they send me to a hundred other things and I'm only spread so thin … That's been the challenge. It's like, "You do this, this, this," and I'm just like, "Well, what's our focus? |
| PACE characteristics and processes (Adaptability, perceived benefit and program resource quality/delivery) | Adaptability/flexibility: a choice-based model  *The PACE program permits teachers to choose from a variety of physical activity options (PE, sport, active lessons or energizers) and to schedule these at their discretion. This flexible, choice-based model was appealing for teachers who were able to choose the “best fit” for their unique class and adapt as needed.* | ISC 76 (P): And whereas you've always got the haters that will say, "Well, there's not enough time in the day," being able to incorporate it into the literacy, into the math, into physical activity, I think it's made a difference. And we're breaking those big sessions up with those little energizers.  ISC 122 (P): I think it's pretty good because it's adaptable. Because everyone's so different and everyone teaches so differently to give 150 minutes and it's kind of like you do with what you can. I think it's really good.  ISC 107 (AP): And also that range of options on the GoNoodle site [energizer website]. To be able to choose what your class really like. Because what my class loves the other classes hate.  ISC 82 (AP): We got a lot out of that, a lot of practical games and activities that we could do, ways to integrate it into our literacy and our maths.  PO 01: …we were able to give them strategies on how to, not create extra time, but fit it in to scheduling; fit those PE lessons, the energizers into their scheduling, they're more likely to go, "Well yeah we do have enough time," alongside of all their other key learning areas or what's happening through the day. Or if we do have an extra assembly that's not on the agenda, we can still fit in, say the energizer at 11 o'clock where it is scheduled on the time table. Whereas possibly in the past, it becomes an issue because we're behind now …so our physical activity might get pushed back.  PO 04: I feel like the overall approach [of staff training] was to show how easy it is to get to 150 minutes, but do it in a way where teachers can pick and choose what they're comfortable with in order to make that 150 minutes. So for example, if some teachers are more comfortable with the FMS style assessment, maybe they would lead that. If there was a teacher that had little experience or lacked confidence in delivering PE, then the energizers using the smart board is a really good way to still make the 150 minutes and still support the program. |
|  | Observable benefits of PACE on students  *Staff observations of the immediate positive impact of PACE on student physical activity and psychosocial health outcomes (physical, social, cognitive, behavioural and affective benefits). This provided incentive for teachers to continue to engage in PACE strategies.* | ISC 122 (P): The staff, all of us, I can speak on my behalf but I know from the others that we can see the difference with the kids that have drive and want to keep on doing it because we see that their attention and their engagement is improved so we make sure we include it in our days because we can see the results are positive.  ISC 110 (P): And there was a few [teachers] there that were a bit, they just like value different things …once we started seeing the development of their [student] fundamental movement skills and, you know, kids are able to catch balls and things like that, they were more or less on board.  ISC 107 (AP): I think the importance of their physical movement; I see a difference in my classroom with my current cohort. And I've been teaching for 28 years and I have some cohorts where it's critical for classroom management because of the nature of the kids …So I feel like our focus has shifted from, “yeah this is what we do” to understanding how important it is that we do it. It's been a complete embracing of that understanding that the physical movement is an essential part of learning. |
|  | An engaging innovation via: | |
|  | Experiential learning  *The practical, hands-on training sessions provided to (i) ISC through the one-day workshop, and (ii) all teachers through the one-hour educational outreach, was a positive “kick start” to the program in schools particularly via active participation.* | ISC 122 (P): I found that for me and my colleagues that did that training, we found that really helpful to just sort of see how that is being acted out. And it was a lot of "aha" moments, trust, where we went, "Oh yes, I can see what I can do in my version of that, or do that and also change it and do another thing." So, I think that's essential because then you can actually see it and do it yourself.  ISC 162 (P): The day [workshop] that we did down there and all the staff were up and doing, I think that that is really beneficial …We're always looking at how we get enough math and English, and all of that in. But, being part of it and actually doing it, which is out of your comfort zone, is what kids feel like …And then got us to actually do what we're expecting the kids to do, to understand how to implement it. So for me, I think that's vital.  ISC 66 (AP): Yeah, there was so much in that [workshop] and it was really practical and me, at that time, I wasn't probably an overly sports person at that stage, but I did feel confident that when I left I could teach it because you came away and it was an actual practical workshop.  ISC 107 (AP): I liked the professional development [workshop] that we went to in February. That was a good kick start because then we came back and presented to the whole school …it kick started the whole conversation of energizers and the timetabling.  PO 03: I think just us having like initially that time at the school champion day where they get to start those policies and get to start that scheduling, see what it looks like and be able to take it back to staff and say, "look, this is how we do it, this is how we fit it in our time table, this is what it looks like".  PO 06: And of course, the actual practical activities were by far probably the biggest enabler, being able to demonstrate how it can be used inside and outside the classroom. |
|  | Project officer characteristics  *The characteristics of project officers assisted schools to implement the program, including via support to ISC. The most commonly cited facilitating characteristics were their positive energy, expert knowledge of physical activity, understanding of the school context (previously teachers themselves), and sincere passion for the program content.* | ISC 62 (P): And we also were lucky enough to have [PO] to come in, and he showed how you could do it easily linked to maths and easily get it within your classrooms for other things as well, which was really good. He was really inspirational. His energy was amazing and the staff really liked having him there …when [PO] came with his energy and made it look so simple, a lot of people instantly started doing it.  ISC 122 (P): Yeah, that's always good to have her [PO] there, to know that you can ask her some questions and she could provide me with some answers and provide me with more resources and that sort of thing; take me in the right direction. I didn't need her in my face all the time, which she wasn't. But it's good to know she was there.  ISC 82 (AP): She's [PO] been very helpful. She's been great. Like the checking in, I spoke before about that accountability and it's good to have someone that checks in, so that it's not pushed to the bottom of the list, ensure it's coming up frequently and kept in the forefront.  PO 03: I feel that if they hear it coming from us, they sort of see it as, “this is important. These guys are experts as such, they know what they're talking about. Yeah, this is what we need to do.”  PO 05: …us going in there, having that support of somebody who knows what's going on and knows what to do and can give that guidance is a big help. |
|  | Quality resources    *As intended, PACE resources assisted ISC and teachers with program implementation, however the high quality of the resources (easy to access, document templates provided, high relevance etc.) were emphasized. Each resource was reported by at least one interviewed ISC, however the use of each varied by school. For example, some schools benefit primarily from the equipment pack whereas others did so by the policy template provided on the online portal.* | ISC 110 (P): Yeah they're good, like they've got good ideas and things like that but the professional learning videos are only quite short but it's good. Especially when you want to show the staff because they don't want to sit around for a while, so if it's short and sharp it's always awesome.  ISC 122 (P): Yeah, I think the resource is perfect. They're really good. We can go to them if we ever really need something, so that's really good to have easy access to that …it gave us a lot more tools to be able to do it and just physically not having to go and find the resources.  ISC 107 (AP): …it gives us an avenue to say, "well we'd love to do this but we don't have the resources". Now we don't have that excuse …If you guys came to us and said "you gotta do this, and this, and this" but then there's no other support, we'd go, "good on you …you've left us high and dry so just bugger off."  PO 01: Definitely all the resources that we provide the schools in templates; in the online portal; the equipment pack; the documentation, so the physical activity policy, helping them with the scope and sequence, giving them examples of timetables …they might be getting 100 minutes. But now we can get to 150 minutes with these extra little resources to help us along the way.  PO 03: I find the [online] portal, as much as we look at the stats sometimes and don't see that traffic in and out, you go to schools and you see that champions have downloaded everything off the portal and given it to all of their staff. I think as a resource place the portal has been fantastic.  PO 06: I thought the website [online portal] was fantastic, the way that it was easy to use for teachers because it had been designed by teachers …It was just simple, practical lessons, which I think was great because sometimes teachers can get caught up with complex lesson plans. Whereas the actual lesson plans around energizers and cross-curricular were simple to use and could be accessed easily on the website. |
| & [+] = Implementation facilitator & [-] = Implementation barrier  **-** | | |
| Abbreviations: ISC= In-school champion; PO= Project officer; P=PACE; AP=Adapted PACE | | |
